# Interactions between the gut microbiome, associated metabolites and the manifestation and progression of heart failure with preserved ejection fraction in ZSF1 rats

**DOI:** 10.1186/s12933-024-02398-6

**Published:** 2024-08-14

**Authors:** Salmina J. Guivala, Konrad A. Bode, Jürgen G. Okun, Ece Kartal, Edzard Schwedhelm, Luca V. Pohl, Sarah Werner, Sandra Erbs, Holger Thiele, Petra Büttner

**Affiliations:** 1 Department of Cardiology, Angiology and Pulmonology, University Hospital Heidelberg, Im Neuenheimer Feld 410, 69120 Heidelberg, Germany; 2 Department Molecular Diagnostics, Laboratory Dr. Limbach and Colleagues, Am Breitspiel 15, 69126 Heidelberg, Germany; 3 Division of Neuropediatrics and Metabolic Medicine, Department of General Pediatrics, University Children’s Hospital Heidelberg, Im Neuenheimer Feld 400, 69120 Heidelberg, Germany; 4 Faculty of Medicine, and Heidelberg University Hospital, Institute for Computational Biomedicine, Bioquant, Heidelberg University, Im Neuenheimer Feld 267, 69120 Heidelberg, Germany; 5 Institute of Clinical Pharmacology and Toxicology, University Medical Center Hamburg-Eppendorf, Martinistraße 52, 20246 Hamburg, Germany; 6 Heart Center Leipzig, University of Leipzig, Strümpellstrasse 89, 04289 Leipzig, Germany

**Keywords:** HFpEF, TMAO, Intestinal microbiome, Inflammation, Intestinal barrier, FMO3, ZSF1-rats

## Abstract

**Background:**

Heart failure with preserved ejection fraction (HFpEF) is associated with systemic inflammation, obesity, metabolic syndrome, and gut microbiome changes. Increased trimethylamine-N-oxide (TMAO) levels are predictive for mortality in HFpEF. The TMAO precursor trimethylamine (TMA) is synthesized by the intestinal microbiome, crosses the intestinal barrier and is metabolized to TMAO by hepatic flavin-containing monooxygenases (FMO). The intricate interactions of microbiome alterations and TMAO in relation to HFpEF manifestation and progression are analyzed here.

**Methods:**

Healthy lean (L-ZSF1, n = 12) and obese ZSF1 rats with HFpEF (O-ZSF1, n = 12) were studied. HFpEF was confirmed by transthoracic echocardiography, invasive hemodynamic measurements, and detection of N-terminal pro-brain natriuretic peptide (NT-proBNP). TMAO, carnitine, symmetric dimethylarginine (SDMA), and amino acids were measured using mass-spectrometry. The intestinal epithelial barrier was analyzed by immunohistochemistry, in-vitro impedance measurements and determination of plasma lipopolysaccharide via ELISA. Hepatic FMO3 quantity was determined by Western blot. The fecal microbiome at the age of 8, 13 and 20 weeks was assessed using 16s rRNA amplicon sequencing.

**Results:**

Increased levels of TMAO (+ 54%), carnitine (+ 46%) and the cardiac stress marker NT-proBNP (+ 25%) as well as a pronounced amino acid imbalance were observed in obese rats with HFpEF. SDMA levels in O-ZSF1 were comparable to L-ZSF1, indicating stable kidney function. Anatomy and zonula occludens protein density in the intestinal epithelium remained unchanged, but both impedance measurements and increased levels of LPS indicated an impaired epithelial barrier function. FMO3 was decreased (− 20%) in the enlarged, but histologically normal livers of O-ZSF1. Alpha diversity, as indicated by the Shannon diversity index, was comparable at 8 weeks of age, but decreased by 13 weeks of age, when HFpEF manifests in O-ZSF1. Bray–Curtis dissimilarity (Beta-Diversity) was shown to be effective in differentiating L-ZSF1 from O-ZSF1 at 20 weeks of age. Members of the microbial families *Lactobacillaceae*, *Ruminococcaceae*, *Erysipelotrichaceae* and *Lachnospiraceae* were significantly differentially abundant in O-ZSF1 and L-ZSF1 rats.

**Conclusions:**

In the ZSF1 HFpEF rat model, increased dietary intake is associated with alterations in gut microbiome composition and bacterial metabolites, an impaired intestinal barrier, and changes in pro-inflammatory and health-predictive metabolic profiles. HFpEF as well as its most common comorbidities obesity and metabolic syndrome and the alterations described here evolve in parallel and are likely to be interrelated and mutually reinforcing. Dietary adaption may have a positive impact on all entities.

**Graphical abstract:**

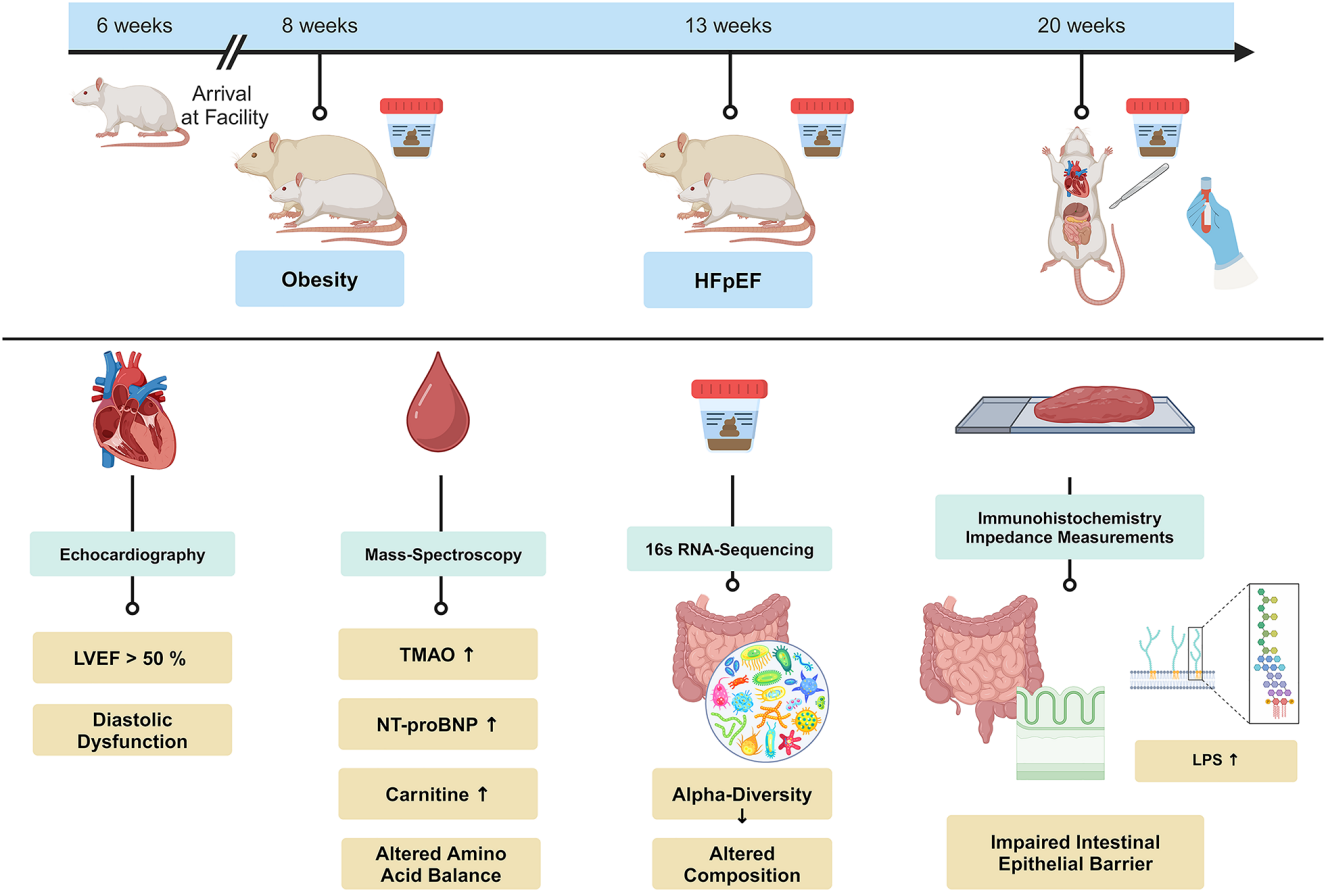

**Supplementary Information:**

The online version contains supplementary material available at 10.1186/s12933-024-02398-6.

## Background

Heart failure (HF) is commonly classified as HF with reduced ejection fraction (HFrEF) or HF with preserved ejection fraction (HFpEF). HFpEF is defined as HF with a left ventricular ejection fraction (LVEF) > 50%, diastolic dysfunction, increased levels of brain natriuretic peptide and/or relevant structural heart disease [1, 2]. HFpEF patients account for 50% of HF patients, yet there are few established prognostically beneficial treatments [1, 3]. While the survival rate for HFrEF has increased over time, that of HFpEF patients has barely been improved. This results in a more severe prognosis, with mortality rates as high as 74% [4]. Various hypotheses have been proposed regarding the underlying pathomechanisms, but it is becoming more evident that HFpEF is linked to metabolic changes and systemic inflammation [5]. To elucidate the pathomechanistic background in HFpEF manifestation robust preclinical models encompassing cofactors and comorbidities are needed [6]. Here, ZSF1-rats, an established HFpEF animal model, were used [7, 8]. Obese ZSF1 rats (O-ZSF1) develop diabetes, dyslipidemia and hypertension, culminating in HFpEF [7–9].

Increased trimethylamine-N-oxide (TMAO) plasma levels have been linked to HFpEF as well as many of its co-morbidities such as atherosclerosis, hypertension, diabetes, and metabolic syndrome [10–15]. A positive correlation between TMAO and indices of diastolic dysfunction, especially mitral and septal wall movement and left atrial volume index was described [12]. TMAO is also associated with significantly higher hospitalization rates, renal dysfunction and cardiac death [16]. It enhances atherosclerosis, promotes thrombosis potential, vascular inflammation and inflammasome activation [17]. The TMAO precursor and rate-limiting synthesis factor, trimethylamine (TMA), is produced by the gut microbiome from dietary choline, phosphatidylcholine, betaine and L-carnitine [18, 19]. When TMA passes the intestinal barrier into portal blood circulation it is metabolized to TMAO by hepatic flavin monooxygenases (FMO), mainly FMO3 [10]. The absorption of TMA from the gut is increased when permeability of the intestinal barrier is higher [20]. Furthermore, the microbiome and its metabolites are in a close bilateral relationship with the intestinal epithelium [21]. In the context of intestinal barrier dysfunction, an involvement of the zonula occludens (ZO1) proteins was proposed [22]. These proteins, primary components of tight junctions, undergo continuous assembly and disassembly and enable rapid adaptation and preservation of the epithelial integrity while simultaneously facilitating the passage of molecules via the paracellular route [23]. Lipopolysaccharide (LPS) represents the nexus between the microbiome, intestinal epithelial barrier and systemic inflammation in HFpEF. LPS, as an integral part of the outer membrane of gram-negative intestinal bacteria, can be translocated to the circulation as a consequence of intestinal barrier dysfunction. Similar to TMAO, it exerts pro-inflammatory effects and has been linked to HFpEF and multiple of its comorbidities [24, 25]. Most importantly, dysbiosis has also been linked to HFpEF [14, 26] and the gut microbiome as well as its metabolites have been proposed as interventional targets in HFpEF. Research on pathomechanisms in manifestation and progression of HFpEF in humans is currently hampered by many obstacles (difficult diagnosis of early progression stages, inaccessibility of tissue samples, heterogeneity of cohorts). Thus, our project aimed to characterize the changes in HFpEF in the ZSF1 animal model by analyzing gut barrier function, the gastrointestinal microbiome and various metabolites in the trajectory of HFpEF progression.

## Methods

### Animals

All experiments and procedures were performed in accordance with relevant guidelines and regulations and were approved by the local Animal Research Council, University of Leipzig and the Landesbehörde Sachsen (TVV 30/18).

Animals were ordered from Charles River (Indianapolis, USA). ZSF1 rats are the result of cross breeding female Zucker diabetes fatty (ZDF) rats with male spontaneously hypertensive heart failure (SHHF) rats. ZDF and SHHF rats both carry unique leptin receptor mutations. Offspring, which is compound heterozygous for the leptin receptor defect, develops the obese phenotype (O-ZSF1), and eventually HFpEF. Rats with none or one mutant allele are of the lean phenotype (L-ZSF1) and do not develop HFpEF. All animals were female littermates. They were kept in identical conditions under a 12:12 h light/dark cycle and were provided with food and water ad libitum. Standard chow was rich in energy and protein content (5008*, ssniff, Soest, Germany). The chow contains 23% protein and 6.5% fat. Fish meal, porcine fat and meat are part of the recipe (Supplementary Fig. 1). The animals were separated depending on their phenotype (two obese or three lean animals per cage) at the age of 6 weeks in order to avoid microbial translocation through coprophagy. Body weight and food intake were recorded weekly. Noninvasive echocardiography (Vivid-J, GE Healthcare, Chicago, USA) was conducted at 20 weeks of age to confirm HFpEF. Additionally, invasive hemodynamic measurements were used to confirm the diagnosis. Before sacrifice deep anesthesia was achieved by intraperitoneal injection of 5 mg/kg xylazine hydrochloride, 100 mg/kg ketamine hydrochloride and 0.1 mg/kg atropine sulfate, based on the individual body weight and animals were killed by exsanguination and immediately dissected.

### Sample processing

Samples of liver and colon located next to caecum were excised and washed in phosphate buffered saline. Colon samples were flash frozen in liquid nitrogen or fixed in 4% phosphate buffered paraformaldehyde. Fecal samples were taken at 8 (baseline), 13 and 20 weeks of age. Baseline samples were collected from the cages without individual assignability, whereas all subsequent samples were obtained individually while animals were under temporary inhalation anesthesia for echocardiographic examination. All samples were stored at −80 °C.

### Determination of TMAO, NT-proBNP, LPS, carnitine, amino acids and SDMA

TMAO was measured using electrospray ionization tandem mass spectrometry according to a modified protocol of Wang et al. as described in Schneider et al. [27]. In brief, a 10 µL plasma sample was pipetted into a 1.5 mL centrifuge tube. After adding 340 µL 4 °C cold mixture of methanol and acetonitrile (ACN) (25:75, v/v) and 50 µL of a 2 µM D_9_-trimethylamine-N-oxide (Cambridge Isotopes Laboratories, Tewksbury, MA, USA) solution in methanol, the protein was precipitated by vortex-mixing for 30 s followed by 10 min incubation. The mixture was centrifuged for 5 min at 18,000×*g* and 150 µL of the supernatant was transferred to a 96-well microplate that was sealed with a preslit adhesive foil. Samples were quantified by an external 9-point calibration using the peak area ratio of TMAO towards D_9_-TMAO. Three quality controls (3 µM, 15 µM, and 75 µM TMAO in fetal calf serum (FCS)) were included in each sample sequence. Liquid chromatography-tandem mass spectrometry (LC-MS/MS) analyses were performed using a Waters XEVO TQS system (Waters, Eschborn, Germany) equipped with an electrospray ion source. The instrument was controlled with MassLynx 4.1 (Waters Corporation, Milford, MA, USA) software. For chromatographic separation, a hydrophilic interaction chromatography column (Waters Acquity UPLC BEH Amide 100 × 2.1 mm; 1.7 μm) with a corresponding pre-column (Waters Acquity UPLC BEH Amide VanGuard, 5 × 2.1 mm; 1.7 µM) was used in isocratic mode. During a 3 min chromatographic run, eluent A (10 mM ammonium formate in ultrapure water (H_2_OmQoutnd ACNout_2_O mQ: ACN 95:5, v/v)) and eluent B (ACN) were applied with a mixing ration of 42% A and 58% B at a flow rate of 0.4 mL/min. The injection volume was 1 µL. The analytes were assessed using a multiple reaction monitoring (MRM) experiment containing their most abundant mass transitions: (TMAO: 76.1 Da → 59.1 Da; d_9_-TMAO: 85.1 Da → 68.1 Da; cone voltage: 40 V; collision energy: 11 V) in positive ion mode at a flow rate of 0.4 ml/min for 3 min.

Established and validated protocols for LC–MS/MS were used to assess SDMA as published before [28]. Briefly, 25 µL of serum were diluted in methanol that contained the stable isotope labeled internal standards. Thereafter, the analytes were converted into their butyl esters. Analyte concentrations were calculated using calibration curves based on four levels in triplicates. Plate wise quality controls were run in two levels by triplicates. A second analysis was done on the samples to assess coefficient of variation and bias of quality control samples, which was below 15% for all analytes.

NT-proBNP was determined in undiluted serum using an ELISA assay according to the manufacturer’s recommendations (abx576280, Hölzel Diagnostika, Cologne, Germany).

LPS was determined using an ELISA according to the manufacturer’s recommendations (CSB-E09945h, Cusabio, Houston, Germany). Due to sample limitations only ten O-ZSF1 and eleven L-ZSF1 rats were accessible for LPS measurements.

For the measurement of the amino acids and the acylcarnitines a 4.7 mm disk was punched out of a blank filter card (Whatman 903 paper) in a 96-well-filterplate. A total of 5 µL plasma was applied and dried overnight at room temperature. The MassChrom^®^ Kit for analysis of amino acids and acylcarnitines from dried blood for newborn screening (57000 F, non-derivatized, Chromsystems Instrument and Chemicals GmbH, Graefelfing, Germany) was used with the following steps: 150 µL of a dilution of the Internal Standards (Internal Standard—Succinylacetone:internal Standard, 1:1, v:v) and 75 µL of the Extraction Buffer—Succinylacetone were added onto the disk. The analytes were extracted by 30 min incubation at 45 °C and 600 rpm on a thermoshaker (Bio-Rad Laboratories GmbH, Feldkirchen, Germany). After centrifuging at 3200*g* for 2 min in a 96-wellplate (V-bottom), 10 µL of the supernatant was injected into the MS/MS system via flow-injection (FIA-MS/MS). Amino acids and acylcarnitines were determined in plasma by electrospray ionization tandem mass spectrometry (ESI-MS/MS) using a Waters Xevo TQD triple quadrupole mass spectrometer (Waters GmbH, Eschborn, Germany) equipped with an electrospray ion source and a Micromass MassLynx data system.

### Western blot analysis

For protein analysis 20 mg of frozen liver samples were homogenized in RIPA buffer containing a protease and a phosphatase inhibitor mix (Serva, Heidelberg, Germany) and sonicated. Protein concentration was determined using the BCA method (bicinchoninic acid assay, Pierce, Bonn, Germany). Antibodies were purchased from Abcam (Cambridge, UK) FMO3 (ab126711) and alpha-Tubulin (ab7291). 25 µg protein were analyzed and FMO3 concentration was normalized to the alpha-tubulin quantity.

### Determination of microbiome

Microbial DNA was extracted from fecal samples using stool transport and recovery (STAR) buffer (Roche, Basel, Switzerland) and SpheroLyse solution (Hain Lifescience, Nehren, Germany). Frozen faeces were thawn, added to the mixture, vortexed until fully blended and incubated at 95 °C for 5 min. Then 50 µL lysozym (4 mg/mL) and lysostaphin (0.01 mg/mL) were added and incubated while shaking at 1200 rpm at 37 °C. Afterwards samples were centrifuged at 5000*g* for 4 min and 100 µL of supernatant was transferred and mixed with 250 µL of STAR-buffer. MagNA Pure 96 standard kit (Roche, Basel, Switzerland) was used for DNA extraction according to the manufacturer’s recommendations. Hypervariable microbial 16s rRNA regions V3 and V4 were identified by PCR using the primers (341F: CCTACGGGNGGCWGCAG and 805R: GACTACHVGGGTATCTAATCC, taken from the manual “16S Metagenomic Sequencing Library Preparation”, Illumina, San Diego US). The Microbial Genomics Module of Qiagen CLC Workbench 12 was used for data analysis. Tables containing operational taxonomic units, closely related sequences of organisms based on a specific similarity threshold 97% were derived by using the Greengenes database (https://greengenes.secondgenome.com/).

### In vitro characterization of intestinal barrier

The barrier function of the gut epithelial cell layer was assessed using epithelial cells, that were isolated from fresh colon samples. About 6 cm of colon were used for cell isolation. Tissue was rinsed with ice-cold phosphate-based saline (PBS) to remove feces and then digested in Dulbecco’s Modified Eagle Medium (DMEM) containing 10% FCS, 10 mg/100 ml collagenase A, 2 mg/100 ml Dispase II (both Roche/Sigma-Aldrich, Taufkirchen, Germany) and 7.7 mg/100 ml 1,4-Dithiothreit (Carl-Roth, Karlsruhe, Germany) for 10 min at 37 °C. Cells were separated from tissue by vigorously shaking, remaining tissue was removed and the cells were pelleted by centrifugation (1000*g*, 10 min, 4 °C). Cells were washed twice in PBS containing 10% FCS and finally digested in Hanks’ Balanced Salt Solution (HBSS) containing 100 mg Dispase II/100 ml under repeated shaking for 10 min at 37 °C. Digestion was stopped by the addition of 10% FCS and cells were passed through a 70 µM and then a 40 µM cell strainer to remove tissue remains. Finally, the cells were washed once with HBSS containing 10% FCS. Cells were cultivated in EBM2 medium (Lonza, Basel, Switzerland) containing 10% FCS, 1% penicillin/streptomycin solution and 1% amphotericin B (both from Sigma-Aldrich, Taufkirchen, Germany) in dishes coated with collagen A (20 µg/ml) (Biochrom, Berlin, Germany) at 37 °C and 7.5% CO_2_. Medium was changed every other day until cells reached 70% confluence when they were passaged three to five times before impedance measurements were done. 5000 cells/well were seeded in cell impedance measurement plates (ACEA Biosciences, San Diego, US) and cell impedance was continuously determined using the xCELLigence Real Time Cell Analyzer system (OLS OMNI Life Science, Bremen, Germany). Once cells reached confluency, represented by an impedance plateau, we started barrier assays by changing 50% of the media to EBM2 medium containing 200 μM histamine or 2% ethanol resulting in a final concentration of 100 µM histamine and 1% ethanol. Following the addition of substances, the decrease in impedance during 30 min was continuously measured. Cell impedance was normalized to the value measured before test substance was added.

### Immunohistochemistry and quantification of ZO1

Colon and liver samples were formaline-fixed, embedded in paraffine and sliced at 2 μm and 5 μm thickness respectively using a manual microtome.

Liver samples were stained with hematoxylin-eosin staining to determine fatty degradation and general morphology with picro sirius red staining to detect collagen and thus fibrosis as recently described [8]. Periodic acid shift (PAS) reaction staining kit (Morphisto, Offenbach am Main, Germany) was used to visualize glycogen according to the manufacturers recommendations (Supplementary Fig. 3).

Colon samples were heated in 10 mM Citrate buffer (pH 6) for 15 min and then were incubated with Proteinase K (Sigma-Aldrich, St. Louis, USA, 20 mg/mL) diluted 1:1000 at 37 °C for 20 min. Blocking was done using 5% horse serum for 30 min. ZO1 was detected using ZO1 rabbit polyclonal antibodies (#61-7300, ThermoFisher, Waltham, USA) at a concentration of 5 µg/mL overnight. Then Goat-anti-Rabbit-IgG secondary antibody labelled with Alexa Fluor 488 (#A11008, ThermoFisher) was added at a concentration of 4 µg/mL for an hour. Hoechst 33342 (ThermoFisher) was used to stain cell nuclei and to facilitate the identification of colon structures. Samples were preserved using anti-fade mounting medium Roti Fluor (ThermoFisher) and were stored at 4 °C in the dark until visualization. Pictures were obtained within 24 h using a Keyence BZ-X810 fluorescence microscope (Keyence, Osaka, Japan).

Images were imported to and analyzed in Fiji/ ImageJ [24]. The outer layers of the intestine, namely tunica submucosa and tunica muscularis were only partially preserved, and therefore excluded from evaluation. The height of the mucosa was measured from the lamina muscularis mucosae to the top of the lamina epithelialis mucosae (five lean and five obese ZSF1 rats, three cross-sections per animal with 20 measurements each). Two to three images at x40 magnification were picked for the analysis of ZO1 antibody staining (see Supplementary Fig. 4). These images were converted into 8-bit format by splitting color channels. Non-destructive contrast improvement was automatically performed for the green channel using the command “Adjust Brightness and Contrast”. Background noise was subtracted using “Subtract Background” with an empirically determined rolling ball radius of 50 pixels. Afterwards cross-sections of intestinal crypts and their barrier to the lumen were identified and circled as regions of interest (ROI). The mean gray value (MGV) per mm^2^ in each ROI—five per image—was measured to indicate the immunofluorescence of the samples.

### Statistical analysis and visualization

Echocardiographic and individual characteristics, TMAO, NT-pro-BNP, LPS, SDMA, and free carnitine are reported as means ± standard deviation. Analysis was done using an unpaired, non-parametric Wilcoxon’s test (Mann–Whitney-U-test) if data was not normally distributed and unpaired t-test with Welch’s correction if normally distributed. Correlations were assessed using the Spearman correlation coefficient (GraphPad Prism^®^ Version 10.0.0, GraphPad Software, Boston, US). Impedance measurements over time were modelled as second order polynomial nonlinear regression and calculated fits were tested for similarity whereas *p* < 0.05 indicated that time lines are different. Protein quantity of FMO3 was normalized to the quantity determined for α-Tubulin and differences between the experimental groups were analyzed using Wilcoxon matched-pairs signed rank test. Microbiome Analysis was performed in R-Studio Version 2023.6.1.524. Microbial composition was analysed on the phylum and family level using the phyloseq and microbiome packages [29, 30]. For this purpose, taxa with low abundance or prevalence were aggregated on the phylum or family level using a detection threshold of 0.001 or 0.01 and prevalence threshold of 0.01 or 0.5 respectively.

Diversity parameters were calculated after rarefaction based on the minimal number of reads across all samples was performed. Alpha-diversity, defined by the Operational Taxonomic Unit (OTU)-based Shannon-Index and richness, was calculated using the vegan package and significance at different time points tested by an unpaired, non-parametric Wilcoxon’s test (Mann–Whitney-U-Test) [31]. The same package was used to assess beta-diversity using the Bray–Curtis dissimilarity index and significance tested by using the adonis2 function (PERMANOVA). Differential Abundance Analysis was performed using the DESeq2 package after pruning all taxa with zero counts in more than 90% o the samples [32]. The log2fold-change describes the ratio of the mean expression level of microbial genes in different conditions. *p* values were adjusted for multiple testing applying the Benjamini-Hochberg procedure and adjusted *p* values were reported. Microbiome analysis was visualized using ggplot2, cowplot and gridExtra (see supplement for further information on the packages used) [33–35]. Furthermore adjusted *p* values ≤ 0.05 were considered statistically significant.

## Results

### Obese ZSF1 rats and lean ZSF1 control rats

Throughout the experimental observation time O-ZSF1 rats constantly had a ~ 30% higher food intake than L-ZSF1. This resulted in significantly higher body weight at the age of 20 weeks (L-ZSF1 235 ± 9 g vs. O-ZSF1 468 ± 24 g, *p* < 0.0001) (Supplementary Fig. 2). Tibia length, a measure of longitudinal growth, was comparable (L-ZSF1 37.1 ± 1.0 mm vs. O-ZSF1 37 ± 0.5 mm, *p* = 0.642). LVEF was similar in L-ZSF1 (55 ± 12%) and O-ZSF1 (63 ± 15%, *p* = 0.140) but an increase in E/e′ ratio, as a measure of myocardial stiffness, was observed in O-ZSF1 (L-ZSF1 15.0 ± 2.8 vs. O-ZSF1 21.7 ± 3.6, *p* < 0.001) (Supplementary Table 1). Furthermore, there was a thickening of the left ventricular wall (L-ZSF1 10 ± 1.5 mm vs. O-ZSF1 12 ± 1.4 mm, *p* = 0.067) and septum (L-ZSF1 7.7 ± 1.2 mm vs. O-ZSF1 8.9 ± 1.2 mm, *p* = 0.044) in obese animals. Livers of O-ZSF1 (18.2 ± 1.9 g) were significantly heavier than those of L-ZSF1 (7.2 ± 0.6 g, *p* < 0.0001).

### Level of circulating TMAO, carnitine, SDMA, amino acids, NT-pro-BNP, LPS, and hepatic FMO3 content

**Fig. 1:**
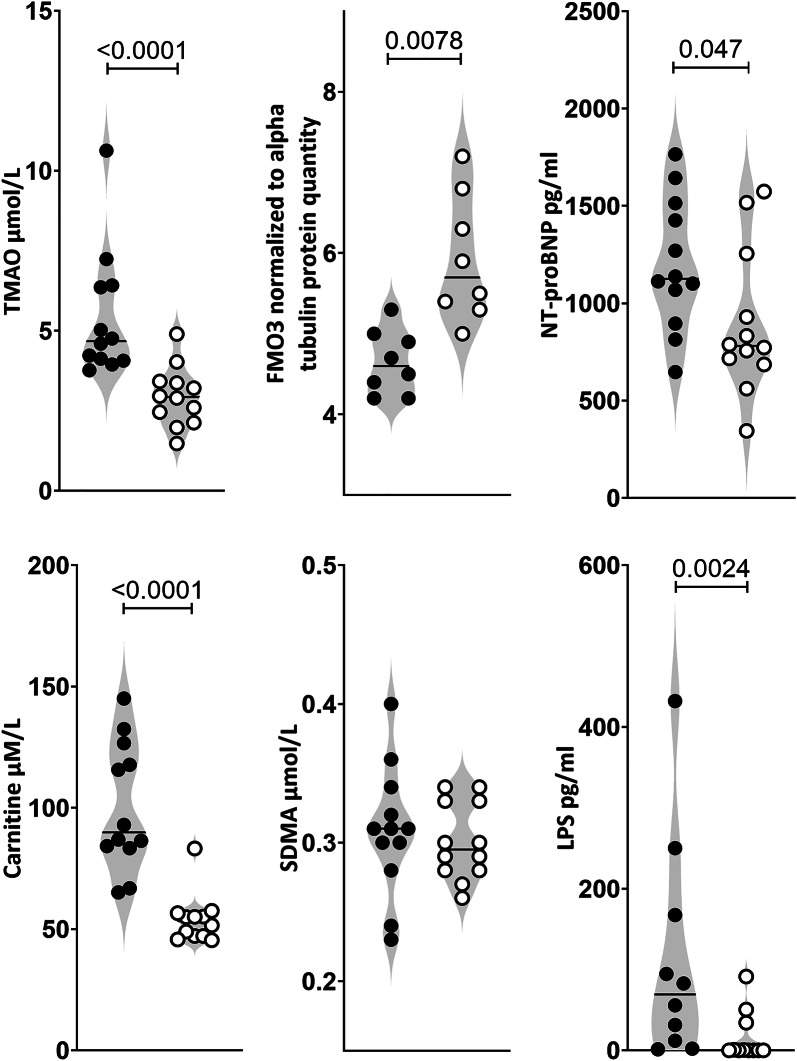
TMAO, TMA-processing flavin containing dimethylaniline monoxygenase 3 (FMO3), NT-proBNP, carnitine, symmetric dimethylarginine (SDMA) and lipopolysaccharide (LPS) quantities in blood/liver of obese (black circles) ZSF1 rats with HFpEF and lean control rats (white circles) (experimental groups *n* = 12, except FMO3 5 vs. 5 and LPS 10 vs. 11 due to limited sample availability). Lines indicate the median

TMAO plasma levels were significantly lower in L-ZSF1 (3.0 ± 0.9 µmol/L, *n* = 12) compared to O-ZSF1 rats (5.5 ± 2.1 µmol/L, *n* = 11; *p* < 0.0001). Overall carnitine was significantly higher in O-ZSF1 (100.3 ± 26.3 µmol/L, *n* = 12) compared to L-ZSF1 rats (54.1 ± 10.2 µmol/L, *n* = 12; *p* < 0.0001). FMO3 expression was significantly > 20% lower in the livers of O-ZSF1 compared to L-ZSF1 (*p* = 0.008). There was no difference in SDMA blood levels in O- and L-ZSF1 (0.301 ± 0.028 µM and 0.308 ± 0.047 µM, *p* = 1.00) (Fig. 1). A total of 17 amino acids were measured in serum, with 14 being higher in O-ZSF1 and 3 being higher in L-ZSF1 (all *p* < 0.05) (Table 1).

**Table 1:** Amino acid level (µmol/L) in plasma of obese (O-ZSF1) and lean (L-ZSF1) rats at an age of 20 weeks

Analyte	O-ZSF1 (mean ± STD)	L-ZSF1 (mean ± STD)	*p* value
Alanine	**344 ± 61**	206 ± 41	< 0.00001
Arginine	60.3 ± 39.7	**148.5 ± 32.2**	0.00001
Aspartic acid	**36.6 ± 8.1**	24.9 ± 4.2	0.00038
Threonine	**24.6 ± 4.7**	17.5 ± 2.8	0.00027
Methionine	**28 ± 5.1**	23.7 ± 3.3	0.02468
Glycine	55.4 ± 9.1	**91.6 ± 11.2**	< 0.00001
Tyrosine	**83.2 ± 17.6**	63.2 ± 15.8	0.00771
Phenylalanine	**88.8 ± 9.4**	78.4 ± 11.2	0.02161
Citrulline	**90.9 ± 9.6**	82.6 ± 10.5	0.05628
Glutamic acid	**99.1 ± 26.2**	53.1 ± 10.2	0.00005
Proline	**142 ± 22**	102 ± 14	0.00004
Ornithine	**230 ± 41**	145 ± 32	0.00001
Valine	**433 ± 32**	274 ± 40	< 0.00001
Tryptophane	**607 ± 66**	520 ± 47	0.00146
Leucine/ Isoleucine	**650 ± 32**	444 ± 68	< 0.00001
Histidine	**1024 ± 146**	908 ± 103	0.0358
Glutamine	4464 ± 605	**5671 ± 915**	0.00117

Bold amino acids are significantly higher in this experimental group

NT-proBNP levels were higher in O-ZSF1 compared to L-ZSF1 rats (1200 ± 338 pg/ml vs. 895 ± 371 pg/ml, *p* = 0.047) (Fig. 1). LPS was significantly higher in O-ZSF1 compared to L-ZSF1 rats (113 ± 137 pg/ml vs. 16 ± 30 pg/ml, *p* = 0.0024) (Fig. 1). Plasma levels of TMAO and it’s substrate, free carnitine, were correlated (*r* = 0.593, *p* = 0.002). NT-pro-BNP and TMAO levels were moderately but significantly correlated (*r* = 0.524, *p* = 0.010). LPS and TMAO levels were moderately but significantly correlated as well (*r* = 0.461, *p* = 0.018).

### In vitro characterization of intestinal barrier function

**Fig. 2:**
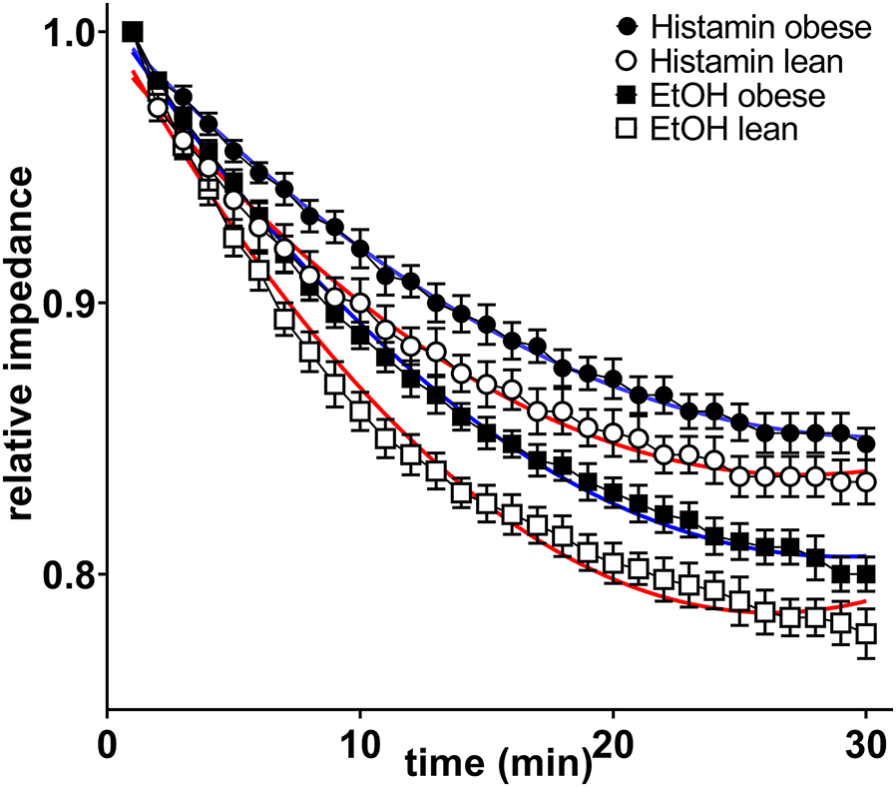
Normalized cellular impedance of colon epithelial cells from obese (black circles and boxes) ZSF1 rats with HFpEF and lean control rats (white circles and boxes) that were exposed to 100 µM histamine (circles) or 1% ethanol (squares) to test the epithelial barrier function. Non-linear fitting curves were added (blue = obese animals, red = lean animals). They differ significantly between both experimental groups (*p* < 0.0001 for histamine and EtOH)

Due to insufficient isolation of viable cells and low proliferative capacity, cells from only five animals per experimental group were available for the final analysis. The introduction of 100 µM histamine and 1% ethanol induced significant, yet reversible reductions in cellular impedance across epithelial cell cultures from all tested animals. However, reversible impedance decrease, as a measure of physiological epithelial barrier function, was significantly less pronounced in cells from O-ZSF1 than in cells from L-ZSF1 (both *p* < 0.0001) (Fig. 2).

### Histological liver characterization

The livers of all animals showed a typical anatomy without any signs of necrosis. No fatty tissue was detected in L-ZSF1 and only two O-ZSF1 had marginal fatty areas < 1%. There were no signs of fibrosis in either group. Glycogen was increased in livers from O-ZSF1 (O-ZSF1 39 ± 13%, L-ZSF1 28 ± 6%, *p* = 0.121) (see Supplementary Fig. 3).

### Histological gut characterization

**Fig. 3:**
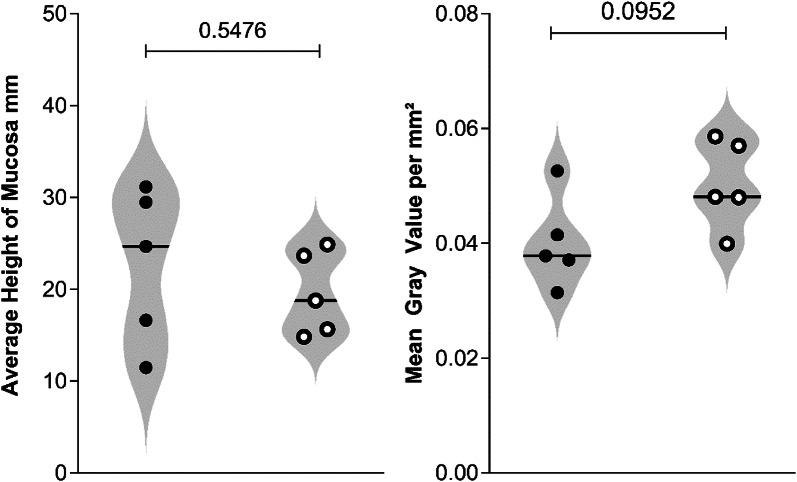
Average Height of Mucosa in cross-sections of colon samples from obese (black cicrles) ZSF1 rats and lean control rats (white circles). Average Mean Gray Value per mm^2^ in areas of interest in colon samples stained with ZO1-antibodies

O-ZSF1 and L-ZSF1 rats showed typically structured intestinal mucosa without anomalies. There was no significant difference in intestinal mucosa height (O-ZSF1 22.87 ± 8.84 mm, L-ZSF1 19.55 ± 5.50 mm, *p* = 0.548) (Fig. 3). Macroscopically, there were no differences in the distribution, composition or arrangement of ZO1 between O-ZSF1 and L-ZSF1. Furthermore, mean gray value per mm^2^, indicating the immunofluorescence signal from regions of interest, were comparable in L-ZSF1 (0.05 ± 0.0025) and O-ZSF1 rats (0.04 ± 0.0035, *p* = 0.095) (Fig. 3).

### Microbiome

**Fig. 4:**
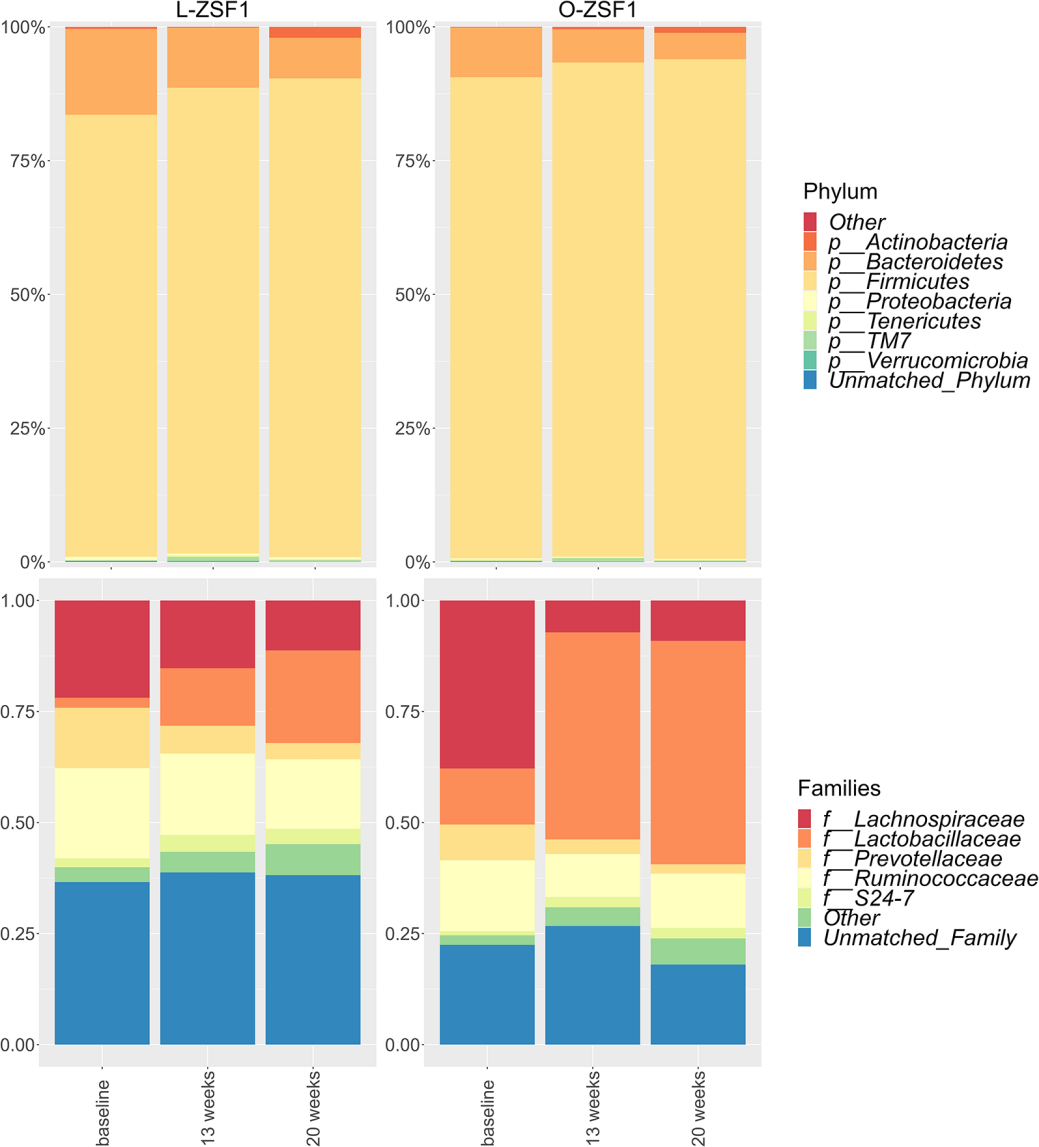
Phylum-level core microbiome composition averaged over time in lean and obese animals at eight, 13 and 20 weeks using relative abundance of phylae. Taxa were aggregated on the phylum-level using a detection threshold of 0.001 and a prevalence threshold of 0.1. Family-level core microbiome composition averaged over time in lean and obese animals at eight, 13 and 20 weeks using relative abundance of families and portraying the top five most prevalent families. Taxa were aggregated on the family-level using a detection threshold of 0.01 and a prevalence threshold of 0.5

**Fig. 5:**
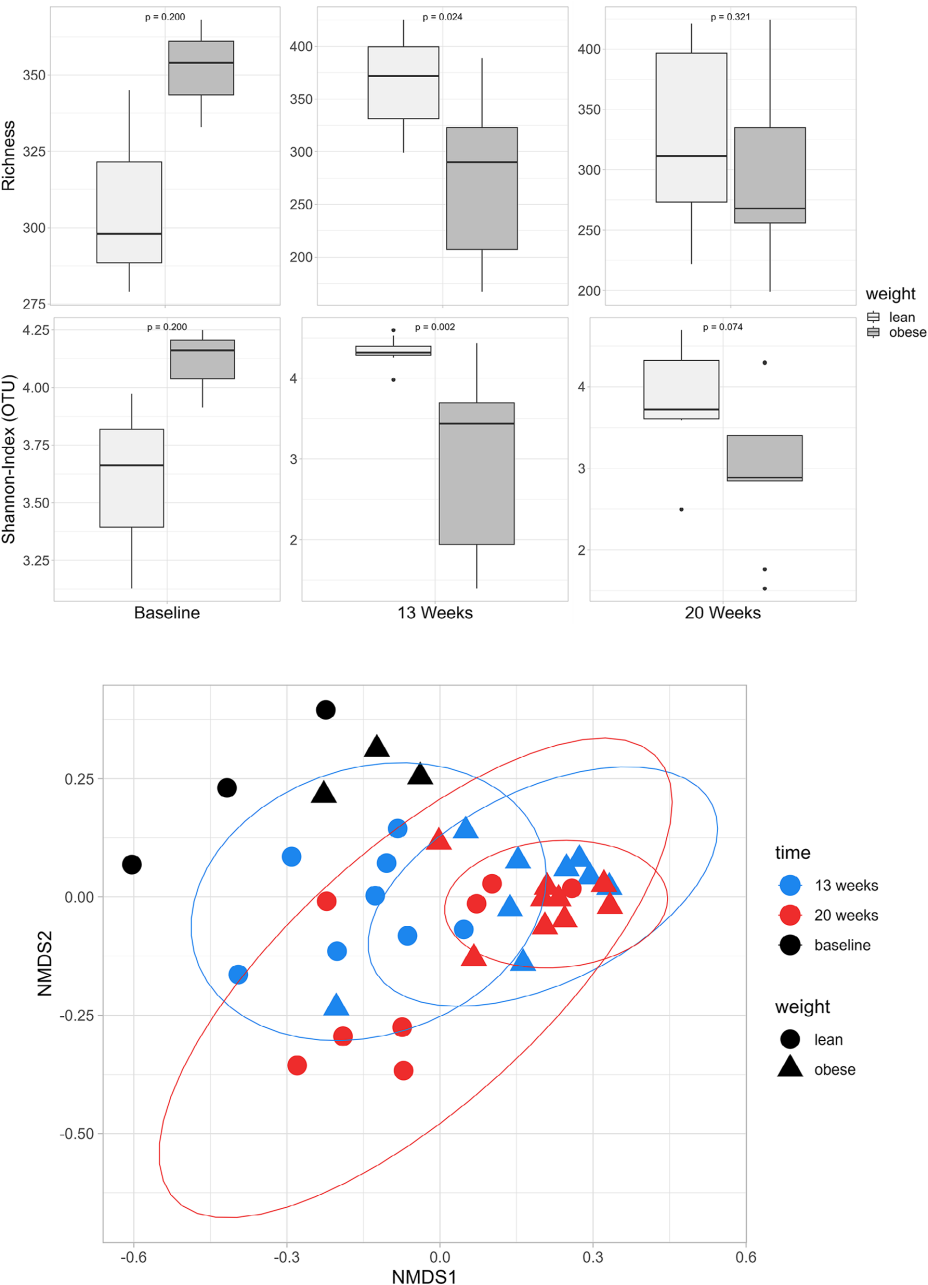
Top: Alpha-diversity represented by Shannon-Index and richness in obese and lean ZSF1 rats at 8, 13 and 20 weeks of age. Lines indicate the median. Bottom: Beta-Diversity in lean versus obese ZSF1-rats at baseline (*p* = 0.200), 13 weeks (*p* = 0.001) und 20 weeks (*p* = 0.007). Non-metric multidimensional scaling (NMDS) was used to illustrate dissimilarities between samples. Ellipses used to show significant grouping. Diversity parameters were analyzed after rarefaction

At an age of 20 weeks the main bacterial phyla in both lean and obese ZSF1 rats were *Firmicutes*, *Bacteroidetes*, *Actinobacteria*, *Proteobacteria* and *Tenericutes* while the most commonly detected families were *Lachnospiriceae*, *Lactobacillaceae*, *Prevotellaceae* and *Ruminococcaceae* (Fig. 4, see Supplementary Fig. 5 for individual samples). At 8 weeks of age, Shannon diversity index (within sample) was comparable (*p* = 0.200), but decreased in O-ZSF1 at 13 weeks, when HFpEF had manifested, and remained lower until the rats were sacrificed at 20 weeks (*p* = 0.003 and *p* = 0.074) (Fig. 5). There was no significant difference in richness, however there was a trend towards a decrease in richness in obese versus lean animals over time (*p* = 0.20, *p* = 0.024 and *p* = 0.320) (Fig. 5). Also, the Bray–Curtis dissimilarity index, representing Beta-diversity, detected no clustering at 8 weeks of age (*p* = 0.200) while at 13 and 20 weeks of age a significant separation of L-ZSF1 and O-ZSF1 was observed (*p* = 0.001 and *p* = 0.007) (Fig. 5). At 20 weeks of age, differential abundance analysis identified 53 strains with significant differences in abundance between O-ZSF1 and L-ZSF1 (α = 0.05, see Supplementary Fig. 6), 21 of which could be identified down to the genus level (Supplementary Table 2). Notably, the abundance of five different *Lactobacillus* species as well as one strain of *Blautia* and *Allobaculum* was decreased while the abundance of five *Oscillospira* species increased in O-ZSF1 (*p* < 0.05).

## Discussion

HFpEF is regarded a multiorgane syndrome with the heart being affected secondary to numerous comorbidities and pathomechanisms [36, 37]. In order to elucidate this multifaceted clinical syndrome, complex pre-clinical models approximating the human reality are needed. ZSF-1 rats cover metabolic and hypertensive stress as well as endothelial dysfunction and systemic inflammation as underpinning pathomechanisms [5, 6, 38–40]. Dysbiosis can aggravate inflammation and is thus supposed to be contributing to the development of HFpEF [14]. The human microbiome is shaped by a plethora of individual or environmental factors like diet, lifestyle and the individual’s environment, including but not limited to vegetable and meat intake, alcohol and nicotine consumption, medication, living region, cleaning habits and household pets [14, 41]. While microbiome analysis in humans is complexified by the numerous influencing factors, an animal model offers the possibility to keep living conditions constant and reduce confounders. We analyzed the role of the gut microbiome and it’s metabolites in an animal model during the manifestation and progression of HFpEF. The diagnosis and progression of HFpEF is best monitored multimodally, combining symptoms, echocardiography, NT-proBNP and invasive hemodynamic measurements. Unfortunately, in rats the latter can only be done before sacrifice. Here echocardiographic data as well as NT-proBNP were only analyzed at an age of 20 weeks. Nevertheless, it was demonstrated in O-ZSF1 rats that E/e′, an early sign of diastolic dysfunction, is detectable already at 10 weeks of age. At the age of 20 weeks, fulminant echocardiographic and invasive signs of diastolic dysfunction; namely anterior wall thickness, end-diastolic diameter and end-diastolic pressure, are present and deterioration continues until 32 weeks of age [8, 39, 42].

The animals were fed a high-energy, high-fat standard diet with porcine meat serving as a dietary source of TMAO precursors. Noteworthy, TMAO and its precursors are natural components of fish and fish-intake was found to increase TMAO levels in humans [15, 19]. Whereas the food intake was increased by ~ 30% in O-SF1, carnitine plasma level and TMAO were increased by 54% compaed to L-ZSF1. Thus it can be inferred that the increased values are due to both increased food intake and intrinsic production. TMAO was shown to be an independent HFpEF risk factor in humans where it is also correlated with NT-proBNP [13, 15]. This correlation was reproduced in our animal model. Interestingly, higher carnitine level, as observed in O-ZSF1, are associated with an increased risk of prevalent coronary and peripheral artery disease, overall cardiovascular disease and 3 year risk for a composite of death, myocardial infarction, stroke, and need of revascularization in humans [43].

TMAO levels are influenced by several factors: TMA production from dietary components by the gastrointestinal microbiome, transition from the gut into the circulation, hepatic metabolization of TMA to TMAO and renal elimination [15]. It was shown that HFpEF and renal dysfunction are closely related with plasma TMAO levels [44]. SDMA is an established marker of kidney function in both rats and human [45–47]. SDMA levels in O-ZSF1 are comparable to L-ZSF1 rats at an age of 20 weeks, whereas renal failure in O-ZSF1 can be excluded [28]. Obese animals showed substantial enlargement of their livers. However, neither signs of fibrosis nor fatty liver disease were present. Therefore, this observation is most likely attributable to a higher glycogen content. The main TMA metabolizing enzyme FMO3 was found to be increased in insulin-resistent mice and humans [15]. Interestingly, it has been demonstrated that female mice exhibit higher FMO3 activity and TMAO accumulation compared to male mice with similar microbiome signature. This might be an underpinning mechanism in the higher HFpEF incidence in women [43]. However, in our O-ZSF1 rats, FMO3 expression was lower compared to L-ZSF1. We did not assess FMO3 activity or measure the less active enzymes FMO4 and FMO5, as this was outside the study’s scope. Nonetheless, it’s probable that elevated TMAO plasma levels are not a result of increased hepatic TMA turnover or inadequate renal elimination.

On the phylum and genus level the microbiome composition in ZSF1 rats was comparable to that of other rat strains [48]. *Firmicutes*, *Proteobacteria* and *Actinobacteria*, considered main TMA producers, are ubiquitously present in the mammalian microbiome and are sufficient in relatively low abundances [15, 43, 49]. Crucially, germ-free mice are incapable of producing TMAO [43]. Nevertheless, it must be taken into account that it is currently not possible to precisely attribute bacteria to diet-related TMAO production [50]. L-ZSF1 and O-ZSF1 rats were kept together before the experiment started and after being separated into different cages received the same food, water and housing conditions whereas O-ZSF1 rats ate more than L-ZSF1 resulting in higher caloric intake. At 8 weeks of age, both experimental groups displayed similar microbial diversity and did not exhibit any significant grouping based on their microbiome. Noteworthy O-ZSF1 rats were already obese at the age of 6 weeks, prior to separation from L-ZSF1, which excludes obesity as the sole cause of the subsequently observed alterations. As HFpEF began to manifest at 10 to 13 weeks and progressed until rats were sacrificed at 20 weeks of age, alpha-diversity, a measure of species diversity within sample, consistently remained significantly lower. Moreover, beta-diversity, a measure of between sample similarity, revealed distinct groupings within the various microbiome landscapes, even at the initial stages of HFpEF manifestation. Generally, a decrease in microbiome diversity has been a defining parameter of dysbiosis in several cardiometabolic and inflammatory diseases [5, 18, 51]. Specifically, humans with HFpEF have also been shown to have diminished microbiome diversity [52, 53] but it remains to be elucidated whether this is a trigger of HFpEF or collateral damage [14]. Noteworthy, in ZSF1 rats cardiac HFpEF characteristics manifest at an age of 12–13 weeks and the disease progresses until the animals show a reduced health at an age of around 32 weeks [8]. Thus, our data suggest that dysbiosis and HFpEF evolve in parallel and may be intertwined. An altered microbiome can also be observed in obese and insulin-resistent patients without HFpEF, linking two of HFpEF’s comorbidities [17].

At the height of HFpEF manifestation, several members of the microbial families *Lactobacillaceae*,* Ruminococcaceae*,* Erysipelotrichaceae* and *Lachnospiraceae* were significantly altered. Members of the *Lactobacillaceae* family showed a decrease in abundance in O-ZSF1, which is consistent with research in other animal models and humans. Interestingly, *Lactobacillus* as well as *Allobaculum*, another less-researched genus decreased in O-ZSF1, have also been linked with vascular endothelial dysfunction—one of the proposed pathomechanisms for HFpEF [54]. Other species, like *Lactobacillus rhamnosus*, have been found to improve systolic and diastolic left ventricular function following coronary ligation artery in rats [17]. *Lactobacillus reuteri*, which had a lower abundance in HFpEF rats, has also been shown to be able to mitigate cardiac injury in high-fat-induced rat models of obesity [55]. Five different members of the genus *Oscillospira* were found to be increased in O-ZSF1 rats. *Oscillospira* has been of high interest in microbiome research, as they account for a high proportion of the fecal microbiome and have been implicated in the pathogenesis of a multitude of metabolic and inflammatory diseases [56, 57]. While some studies attribute beneficial effects of weight loss and amelioration of metabolic syndrome to *Oscillospira*, an increased abundance has also been linked to a high-fat diet in different rodent models as well as diabetes and inflammation in type 2 diabetes rat models [56, 58]. Here, further research diving into the specific subspecies and strains of Oscillospira is needed. Lastly, *Blautia*, decreased in O-ZSF1 rats, has been linked to not only HF in general but HFpEF specifically [59, 60].

Amino acid levels were also altered in O-ZSF1. This is partly attributable to altered utilization of arginine by arginases and NO synthases with consequentially increased turnover products ornithine and citrulline in this animal model [28, 61]. However, the intestinal microbiome is also involved in the human amino acid homeostasis, and vice versa amino acids have an impact on the abundance of the microbes that utilize them [14]. For example, proline is the preferred amino acid substrate of *Clostridium* genus bacteria, while other anaerobes, including *Bacteroides*, *Lactobacillus*, *Bifidobacterium*, and *Peptostreptococcus* ferment the aromatic amino acids [52] phenylalanine, tryptophan and tyrosine. All these amino acids were higher in O-ZSF1 compared to L-ZSF1 and likewise some of the metabolizing microbes were more abundant in O-ZSF1. Interestingly, high plasma levels of phenylalanine, as observed in the HFpEF rats, are associated with higher levels of C-reactive protein and inflammatory cytokines as well as higher mortality in patients with heart failure [14, 62]. Further, increased levels of leucine, histidine, ornithine and phenylalanine as observed in O-ZSF1 were associated with higher event rates in HF populations [52, 63]. On the other hand, some amino acids also were lower in O-ZSF1 like glycine which was found to have anti-inflammatory effects and a beneficial antihypertrophy effect in heart failure [14, 52]. Noteably, tryptophane, an amino acid involved in complex pathways that are both beneficial and detrimental to cardiovascular health, was increased in obese animals [64, 65].

After TMA is produced in the gut it crosses the intestinal gut barrier and passes over to the blood stream. This process is facilitated when tight junction dysfunction occurs or biological barriers are impaired, a phenomenon that is referred to as leaky gut concept [17]. Gut barrier dysfunction can also lead to the transition of microbial metabolites and endotoxins to the blood stream, where these trigger inflammatory cascades and thus contribute to HFpEF. In turn, HFpEF has been associated with insufficient cardiac output, leading to intestinal ischemia, edema, and inflammation, which consecutively impairs the epithelial barrier function [14]. While the intestines of obese ZSF1 rats showed no major histological changes, the aforementioned effects are mirrored by the observed epithelial barrier dysfunction in diseased animals. Further, elevated levels of LPS, an established marker of increased gut permeability and endotoxin, were observed in O-ZSF1. This is comparable to findings in humans with heart failure [66]. Both TMAO and LPS exert pro-inflammatory effects by activating several intracellular pathways including the NLRP3 inflammasome [17, 67, 68]. TMAO itself has been found to damage epithelial cells and impair their self-repair and thereby influence barrier function [15, 69]. Using the ZSF1 rat model our results demonstrate that changes in the microbiome occur at an early timepoint in HFpEF progression, before cardiac function is distinctively impaired [8]. Thus, alterations in microbiome diversity may initially trigger the development of HFpEF and after onset of the disease intestinal damage might be accelerated in a vicious cycle. In this context, the gastrointestinal microbiome has emerged as an exceptionally modifiable organ that could be the target of low-effort, noninvasive and low-cost interventions [51]. TMA and thus TMAO levels can be influenced by limitation of carnitine and cholin, found highly concentrated in foods from animal origin like meat and dairy products, and representing main sources for bacterial TMA production [15]. However, TMAO is also a stable compound of the mediterranean diet, considered beneficial to cardiovascular health, and it has been reported that TMAO supplementation in hypertensive rats prevented heart failure-associated mortality and had diuretic effects [70]. On the other hand, it was shown that the administration of TMAO has pro-inflammatory, pro-thrombotic and pro-atherogenic effects [71]. In summary, while intrinsic TMAO elevation is associated with unfavorable cardiovascular outcomes, inconsistent results of supplementation studies suggest that artificial elevation of TMAO fails to account for all involved mechanisms [72]. In line with the results of our study, it can be hypothesized that TMAO is not an isolated causative factor, but primarily a surrogate for the highly complex pathomechanisms underlying cardiovascular disease.

A diet rich in plant-based fiber and fermented foods, increases the diversity of the microbiome, decreases inflammatory markers [73] and simultaneously reduces TMAO [43]. In contrast, a high-fat- low-fibre-diet has been linked to dysbiosis, disruption of intestinal permeability as well as an increase in bacteria involved in cardiovascular diseases [51]. Pre- and probiotics or the administration of beneficial microbes may also be useful [17, 51]. For example, the application of *Lactobacillus rhamnosus GR-1* has already been discussed as a therapy for heart failure and the bacteria has even been shown to reduce atherosclerotic plaque size [52]. Moreover, the administration of antibiotics can also decrease TMAO levels and reverse effects of TMAO administration [74]. Finally, exercise was also found to have a beneficial effect on the gut microbial diversity in Wistar rats [56].

## Conclusion

The ZSF1 rat model mirrors many alterations observed in human patients, making it a well-suited choice to investigate the connection between the gut microbiome and its metabolites during the manifestation and progression of HFpEF with minimial influencing factors.

1. The gastrointestinal microbiome changes parallel to HFpEF manifestation and progression and both entities may be the consequences of the same underpinning pathomechanism.
2. The gut epithelial barrier function in O-ZSF1 is impaired. This may cause “intestinal leakage” of microbial metabolites and particles, like TMAO and LPS, into the blood stream, resulting in a pro-inflammatory environment.
3. Despite identical diet composition, free carnitine, the TMA precursor, is higher in the blood of O-ZSF1 due to generally higher food intake. Further, pronounced differences in amino acid levels were observed. This may favour changes in microbiome composition, represent altered microbial metabolite turnover and finally increase adverse systemic effects like inflammation or epithelial dysfunction.

### Limitations

This study is based on a small sample number. Although the ZSF1 rat model mirrors HFpEF characteristics and pathomechanisms observed in humans, it is unclear whether the findings can be transferred. Additionally, the pathophysiology of HFpEF is defined by a manifold of heterogenous mechanisms, which can only partially be covered by an animal model. As current literature suggests differences between sex as a risk factor in pre-clinical and clinical models, the findings of this study should also be evaluated in male animals.

Fecal samples represent the microbiome composition of the colon. A more in-depth analysis should include the extraction of fecal matter from different parts of the intestine as well as luminal and mucosal samples. Using 16s rRNA sequencing from faeces is a reliable, established and inexpensive way of microbiome assessment, however taxonomic resolution and coverage are limited. Furthermore, this method can not directly analyse microbial function. While our analysis was able to partially account for these challenges and is therefore suitable to detect differences between the experimental groups, it was not possible to fully map the ZSF1 microbiome. Unidentified species could pose any form of contribution, harmful or beneficial, to the microbial community or it’s host. In the future, shotgun metagenomic sequencing and additional omics-data will aid in defining not only composition, but also the functional profile of bacteria. To differentiate collateral damage from triggers, further analysis could also include the administration of microbiome-altering substances such as synbiotics or TMAO itself. Immunohistochemistry is at best a semiquantitative method with pronounced inter-observer assessment divergence [75]. Additionally, the intestinal barrier is a complex construct comprised of mucins, antibacterial proteins, epithelial cells and the vascular endothelium. Future research may encompass a more global analysis of the components of intestinal barrier function [22].

### Supplementary material

Supplementary Material 1.

## Data Availability

The datasets used and/or analysed during the current study are available from the corresponding author on reasonable request.

## Abbreviations

ACN: Acetonitrile
DMEM: Dulbecco’s Modified Eagle Medium
E/e′: Ratio of early diastolic maximum transmitral inflow velocity (E) to early diastolic tissue velocity at the mitral valve annulus (e′)
ESI-MS/MS: Electrospray ionization tandem mass spectrometry
FCS: Fetal calf serum
FIA-MS/MS: Flow-injection MS/MS system
FMO3: Flavin-containing mono-oxygenase 3
HBSS: Hanks’ balanced salt solution
HF: Heart failure
HFpEF: Heart failure with preserved ejection fraction
LVEF: Left ventricular ejection fraction
L-ZSF1: Lean ZSF1 rats
MGV: Mean gray value
MRM: Multiple reaction monitoring
MS: Mass spectrometry
NO: Nitric oxide
NT-proBNP: N-terminal pro-brain natriuretic peptide
OTU: Operational taxonomic unit
O-ZSF1: Obese ZSF1 rats
PAS: Periodic acid shift
PBS: Phosphate-based saline
ROI: Region of interest
SDMA: Symmetric dimethylarginine
TMA: Trimethylamine
TMAO: Trimethylamine-N-oxide
ZO1: Zona occludens protein 1
ZSF1: F1 hybrids crossed from Zucker diabetes fatty (ZDF)-rats with male spontaneously hypertensive heart failure (SHHF)-rats

## References

[1] McDonagh TA, Metra M, Adamo M, Gardner RS, Baumbach A, Böhm M, et al. 2023 focused update of the 2021 ESC guidelines for the diagnosis and treatment of acute and chronic heart failure. Eur Heart J. 2023. 10.1093/eurheartj/ehad195. 37622666 10.1093/eurheartj/ehad195

[2] Reddy YNV, Borlaug BA. Heart failure with preserved ejection fraction. Curr Probl Cardiol. 2016;41:145–88. 10.1016/j.cpcardiol.2015.12.002. 26952248 10.1016/j.cpcardiol.2015.12.002

[3] Nassif ME, Windsor SL, Borlaug BA, Kitzman DW, Shah SJ, Tang F, et al. The SGLT2 inhibitor dapagliflozin in heart failure with preserved ejection fraction: a multicenter randomized trial. Nat Med. 2021;27:1954–60. 10.1038/s41591-021-01536-x. 34711976 10.1038/s41591-021-01536-x PMC8604725

[4] Dunlay SM, Roger VL, Redfield MM. Epidemiology of heart failure with preserved ejection fraction. Nat Rev Cardiol. 2017;14:591–602. 10.1038/nrcardio.2017.65. 28492288 10.1038/nrcardio.2017.65

[5] Yoo JY, Sniffen S, McGill Percy KC, Pallaval VB, Chidipi B. Gut dysbiosis and immune system in atherosclerotic cardiovascular disease (ACVD). Microorganisms. 2022. 10.3390/microorganisms10010108. 35056557 10.3390/microorganisms10010108 PMC8780459

[6] Roh J, Hill JA, Singh A, Valero-Muñoz M, Sam F. Heart failure with preserved ejection fraction: heterogeneous syndrome, diverse preclinical models. Circ Res. 2022;130:1906–25. 10.1161/CIRCRESAHA.122.320257. 35679364 10.1161/CIRCRESAHA.122.320257 PMC10035274

[7] Conceição G, Heinonen I, Lourenço AP, Duncker DJ, Falcão-Pires I. Animal models of heart failure with preserved ejection fraction. Neth Heart J. 2016;24:275–86. 10.1007/s12471-016-0815-9. 26936157 10.1007/s12471-016-0815-9 PMC4796054

[8] Schauer A, Draskowski R, Jannasch A, Kirchhoff V, Goto K, Männel A, et al. ZSF1 rat as animal model for HFpEF: development of reduced diastolic function and skeletal muscle dysfunction. ESC Heart Fail. 2020;7:2123–34. 10.1002/ehf2.12915. 32710530 10.1002/ehf2.12915 PMC7524062

[9] Bilan VP, Salah EM, Bastacky S, Jones HB, Mayers RM, Zinker B, et al. Diabetic nephropathy and long-term treatment effects of rosiglitazone and enalapril in obese ZSF1 rats. J Endocrinol. 2011;210:293–308. 10.1530/JOE-11-0122. 21680617 10.1530/JOE-11-0122

[10] Zeisel SH, Warrier M. Trimethylamine N-oxide, the microbiome, and heart and kidney disease. Annu Rev Nutr. 2017;37:157–81. 10.1146/annurev-nutr-071816-064732. 28715991 10.1146/annurev-nutr-071816-064732

[11] Al-Rubaye H, Perfetti G, Kaski J-C. The role of microbiota in cardiovascular risk: focus on trimethylamine oxide. Curr Probl Cardiol. 2019;44:182–96. 10.1016/j.cpcardiol.2018.06.005. 30482503 10.1016/j.cpcardiol.2018.06.005

[12] Tang WHW, Wang Z, Shrestha K, Borowski AG, Wu Y, Troughton RW, et al. Intestinal microbiota-dependent phosphatidylcholine metabolites, diastolic dysfunction, and adverse clinical outcomes in chronic systolic heart failure. J Card Fail. 2015;21:91–6. 10.1016/j.cardfail.2014.11.006. 25459686 10.1016/j.cardfail.2014.11.006 PMC4312712

[13] Dong Z, Zheng S, Shen Z, Luo Y, Hai X. Trimethylamine N-oxide is associated with heart failure risk in patients with preserved ejection fraction. Lab Med. 2021;52:346–51. 10.1093/labmed/lmaa075. 33135738 10.1093/labmed/lmaa075

[14] Yu W, Jiang Y, Xu H, Zhou Y. The Interaction of Gut Microbiota and Heart failure with preserved ejection fraction: from mechanism to potential therapies. Biomedicines. 2023. 10.3390/biomedicines11020442. 36830978 10.3390/biomedicines11020442 PMC9953339

[15] Simó C, García-Cañas V. Dietary bioactive ingredients to modulate the gut microbiota-derived metabolite TMAO. New opportunities for functional food development. Food Funct. 2020;11:6745–76. 10.1039/d0fo01237h. 32686802 10.1039/d0fo01237h

[16] Kinugasa Y, Nakamura K, Kamitani H, Hirai M, Yanagihara K, Kato M, Yamamoto K. Trimethylamine N-oxide and outcomes in patients hospitalized with acute heart failure and preserved ejection fraction. ESC Heart Fail. 2021;8:2103–10. 10.1002/ehf2.13290. 33734604 10.1002/ehf2.13290 PMC8120352

[17] Witkowski M, Weeks TL, Hazen SL. Gut microbiota and cardiovascular disease. Circ Res. 2020;127:553–70. 10.1161/CIRCRESAHA.120.316242. 32762536 10.1161/CIRCRESAHA.120.316242 PMC7416843

[18] Peng J, Xiao X, Hu M, Zhang X. Interaction between gut microbiome and cardiovascular disease. Life Sci. 2018;214:153–7. 10.1016/j.lfs.2018.10.063. 30385177 10.1016/j.lfs.2018.10.063

[19] Evans M, Dai L, Avesani CM, Kublickiene K, Stenvinkel P. The dietary source of trimethylamine N-oxide and clinical outcomes: an unexpected liaison. Clin Kidney J. 2023;16:1804–12. 10.1093/ckj/sfad095. 37915930 10.1093/ckj/sfad095 PMC10616480

[20] Drapala A, Szudzik M, Chabowski D, Mogilnicka I, Jaworska K, Kraszewska K, et al. Heart failure disturbs gut-blood barrier and increases plasma trimethylamine, a toxic bacterial metabolite. Int J Mol Sci. 2020. 10.3390/ijms21176161. 32859047 10.3390/ijms21176161 PMC7504565

[21] Goyal S, Tsang DKL, Maisonneuve C, Girardin SE. Sending signals—the microbiota’s contribution to intestinal epithelial homeostasis. Microbes Infect. 2021;23:104774. 10.1016/j.micinf.2020.10.009. 33189870 10.1016/j.micinf.2020.10.009

[22] Lewis CV, Taylor WR. Intestinal barrier dysfunction as a therapeutic target for cardiovascular disease. Am J Physiol Heart Circ Physiol. 2020;319:H1227–33. 10.1152/ajpheart.00612.2020. 32986965 10.1152/ajpheart.00612.2020 PMC7792706

[23] Kuo W-T, Odenwald MA, Turner JR, Zuo L. Tight junction proteins occludin and ZO-1 as regulators of epithelial proliferation and survival. Ann NY Acad Sci. 2022;1514:21–33. 10.1111/nyas.14798. 35580994 10.1111/nyas.14798 PMC9427709

[24] Ghosh SS, Wang J, Yannie PJ, Ghosh S. Intestinal barrier dysfunction, LPS translocation, and disease development. J Endocr Soc. 2020;4:bvz039. 10.1210/jendso/bvz039. 32099951 10.1210/jendso/bvz039 PMC7033038

[25] Violi F, Castellani V, Menichelli D, Pignatelli P, Pastori D. Gut barrier dysfunction and endotoxemia in heart failure: a dangerous connubium? Am Heart J. 2023;264:40–8. 10.1016/j.ahj.2023.06.002. 37301317 10.1016/j.ahj.2023.06.002

[26] Yu Y, Xiong Y, Montani J-P, Yang Z, Ming X-F. En face detection of nitric oxide and superoxide in endothelial layer of intact arteries. J Vis Exp. 2016;108:53718. 10.3791/53718. 10.3791/53718 PMC4828200 26967197

[27] Wang Z, Levison BS, Hazen JE, Donahue L, Li X-M, Hazen SL. Measurement of trimethylamine-N-oxide by stable isotope dilution liquid chromatography tandem mass spectrometry. Anal Biochem. 2014;455:35–40. 10.1016/j.ab.2014.03.016. 24704102 10.1016/j.ab.2014.03.016 PMC4167037

[28] Büttner P, Werner S, Baskal S, Tsikas D, Adams V, Lurz P, et al. Arginine metabolism and nitric oxide turnover in the ZSF1 animal model for heart failure with preserved ejection fraction. Sci Rep. 2021;11:20684. 10.1038/s41598-021-00216-7. 34667218 10.1038/s41598-021-00216-7 PMC8526609

[29] Ernst FG, Shetty S, Borman T, Braccia DJ, Huang R, Corrada Bravo H, Lahti L. Microbiome @ Git Hub. https://microbiome.github.io/. Accessed 6 Mar 2024.

[30] McMurdie PJ, Holmes S. Phyloseq: an R package for reproducible interactive analysis and graphics of microbiome census data. PLoS ONE. 2013;8:e61217. 10.1371/journal.pone.0061217. 23630581 10.1371/journal.pone.0061217 PMC3632530

[31] Community. Ecology Package [R package vegan version 2.6-4]: Comprehensive R Archive Network (CRAN).

[32] Love MI, Huber W, Anders S. Moderated estimation of Fold change and dispersion for RNA-seq data with DESeq2. Genome Biol. 2014;15:550. 10.1186/s13059-014-0550-8. 25516281 10.1186/s13059-014-0550-8 PMC4302049

[33] Create Elegant Data Visualisations. Using the Grammar of Graphics [R package ggplot2 version 3.5.0]: Comprehensive R Archive Network (CRAN).

[34] Streamlined Plot Theme and Plot Annotations for ‘ggplot2’ [R package cowplot version 1.1.3]: Comprehensive R Archive Network (CRAN); 2024.

[35] Baptiste Auguie. Miscellaneous functions for Grid Graphics [R package gridExtra version 2.3]: Comprehensive R Archive Network. CRAN); 2017.

[36] Mishra S, Kass DA. Cellular and molecular pathobiology of heart failure with preserved ejection fraction. Nat Rev Cardiol. 2021;18:400–23. 10.1038/s41569-020-00480-6. 33432192 10.1038/s41569-020-00480-6 PMC8574228

[37] Triposkiadis F, Butler J, Abboud FM, Armstrong PW, Adamopoulos S, Atherton JJ, et al. The continuous heart failure spectrum: moving beyond an ejection fraction classification. Eur Heart J. 2019;40:2155–63. 10.1093/eurheartj/ehz158. 30957868 10.1093/eurheartj/ehz158 PMC7963129

[38] Ostadal P, Mlcek M, Gorhan H, Simundic I, Strunina S, Hrachovina M, et al. Electrocardiogram-synchronized pulsatile extracorporeal life support preserves left ventricular function and coronary flow in a porcine model of cardiogenic shock. PLoS ONE. 2018;13:e0196321. 10.1371/journal.pone.0196321. 29689088 10.1371/journal.pone.0196321 PMC5915277

[39] Leite S, Oliveira-Pinto J, Tavares-Silva M, Abdellatif M, Fontoura D, Falcão-Pires I, et al. Echocardiography and invasive hemodynamics during stress testing for diagnosis of heart failure with preserved ejection fraction: an experimental study. Am J Physiol Heart Circ Physiol. 2015;308:H1556–63. 10.1152/ajpheart.00076.2015. 25862827 10.1152/ajpheart.00076.2015

[40] van Dijk CGM, Oosterhuis NR, Xu YJ, Brandt M, Paulus WJ, van Heerebeek L, et al. Distinct endothelial cell responses in the heart and kidney microvasculature characterize the progression of heart failure with preserved ejection fraction in the obese ZSF1 rat with Cardiorenal metabolic syndrome. Circ Heart Fail. 2016;9:e002760. 10.1161/CIRCHEARTFAILURE.115.002760. 27056881 10.1161/CIRCHEARTFAILURE.115.002760

[41] Hills RD, Pontefract BA, Mishcon HR, Black CA, Sutton SC, Theberge CR. Gut microbiome: profound implications for diet and disease. Nutrients. 2019. 10.3390/nu11071613. 31315227 10.3390/nu11071613 PMC6682904

[42] Leite S, Cerqueira RJ, Ibarrola J, Fontoura D, Fernández-Celis A, Zannad F, et al. Arterial remodeling and dysfunction in the ZSF1 rat model of heart failure with preserved ejection fraction. Circ Heart Fail. 2019;12:e005596. 10.1161/CIRCHEARTFAILURE.118.005596. 31525070 10.1161/CIRCHEARTFAILURE.118.005596

[43] Koeth RA, Wang Z, Levison BS, Buffa JA, Org E, Sheehy BT, et al. Intestinal microbiota metabolism of L-carnitine, a nutrient in red meat, promotes atherosclerosis. Nat Med. 2013;19:576–85. 10.1038/nm.3145. 23563705 10.1038/nm.3145 PMC3650111

[44] Guo F, Qiu X, Tan Z, Li Z, Ouyang D. Plasma trimethylamine n-oxide is associated with renal function in patients with heart failure with preserved ejection fraction. BMC Cardiovasc Disord. 2020;20:394. 10.1186/s12872-020-01669-w. 32859154 10.1186/s12872-020-01669-w PMC7456383

[45] Hamlin DM, Schultze AE, Coyne MJ, McCrann DJ, Mack R, Drake C et al. Evaluation of renal biomarkers, including symmetric dimethylarginine, following gentamicin-induced proximal tubular injury in the rat. Kidney360. 2022;3:341–56. 10.34067/KID.0006542020 35373128 10.34067/KID.0006542020 PMC8967625

[46] Michael H, Szlosek D, Clements C, Mack R. Symmetrical dimethylarginine: evaluating chronic kidney disease in the era of multiple kidney biomarkers. Vet Clin North Am Small Anim Pract. 2022;52:609–29. 35379500 10.1016/j.cvsm.2022.01.003

[47] Oliva-Damaso E, Oliva-Damaso N, Rodriguez-Esparragon F, Payan J, Baamonde-Laborda E, Gonzalez-Cabrera F, et al. Asymmetric (ADMA) and symmetric (SDMA) dimethylarginines in chronic kidney disease: a clinical approach. Int J Mol Sci. 2019. 10.3390/ijms20153668. 31357472 10.3390/ijms20153668 PMC6696355

[48] Li D, Chen H, Mao B, Yang Q, Zhao J, Gu Z, et al. Microbial biogeography and core microbiota of the rat digestive tract. Sci Rep. 2017;8:45840. 10.1038/srep45840. 28374781 10.1038/srep45840 PMC5379200

[49] Rath S, Rud T, Pieper DH, Vital M. Potential TMA-producing bacteria are ubiquitously found in Mammalia. Front Microbiol. 2019;10:2966. 10.3389/fmicb.2019.02966. 31998260 10.3389/fmicb.2019.02966 PMC6964529

[50] Ferrell M, Bazeley P, Wang Z, Levison BS, Li XS, Jia X, et al. Fecal microbiome composition does not predict diet-induced TMAO production in healthy adults. J Am Heart Assoc. 2021;10:e021934. 10.1161/JAHA.121.021934. 34713713 10.1161/JAHA.121.021934 PMC8751816

[51] Ghosh TS, Valdes AM. Evidence for clinical interventions targeting the gut microbiome in cardiometabolic disease. BMJ. 2023;383:e075180. 10.1136/bmj-2023-075180. 37813434 10.1136/bmj-2023-075180 PMC10561016

[52] Tuerhongjiang G, Guo M, Qiao X, Lou B, Wang C, Wu H, et al. Interplay between gut microbiota and amino acid metabolism in Heart failure. Front Cardiovasc Med. 2021;8:752241. 10.3389/fcvm.2021.752241. 34746265 10.3389/fcvm.2021.752241 PMC8566708

[53] Luedde M, Winkler T, Heinsen F-A, Rühlemann MC, Spehlmann ME, Bajrovic A, et al. Heart failure is associated with depletion of core intestinal microbiota. ESC Heart Fail. 2017;4:282–90. 10.1002/ehf2.12155. 28772054 10.1002/ehf2.12155 PMC5542738

[54] Liu T, Li X, Zhang C, Zhao L, Li X, Yu Y, et al. *Lactobacillus* and *Allobaculum* mediates the improvement of vascular endothelial dysfunction during hypertension with TaohongSiwu decoction combined with Dubosiella newyorkensis. Heliyon. 2023;9:e22572. 10.1016/j.heliyon.2023.e22572. 38089998 10.1016/j.heliyon.2023.e22572 PMC10711123

[55] Liao P-H, Kuo W-W, Hsieh DJ-Y, Yeh Y-L, Day C-H, Chen Y-H, et al. Heat-killed *Lactobacillus reuteri* GMNL-263 prevents epididymal fat accumulation and cardiac injury in high-calorie diet-fed rats. Int J Med Sci. 2016;13:569–77. 10.7150/ijms.15597. 27499689 10.7150/ijms.15597 PMC4974905

[56] Yang J, Li Y, Wen Z, Liu W, Meng L, Huang H. Oscillospira—a candidate for the next-generation probiotics. Gut Microbes. 2021;13:1987783. 10.1080/19490976.2021.1987783. 34693878 10.1080/19490976.2021.1987783 PMC8547878

[57] Wei B, Wang S, Wang Y, Ke S, Jin W, Chen J, et al. Gut microbiota-mediated xanthine metabolism is associated with resistance to high-fat diet-induced obesity. J Nutr Biochem. 2021;88:108533. 10.1016/j.jnutbio.2020.108533. 33250443 10.1016/j.jnutbio.2020.108533

[58] Zhu Y, Dong L, Huang L, Shi Z, Dong J, Yao Y, Shen R. Effects of oat β-glucan, oat resistant starch, and the whole oat flour on insulin resistance, inflammation, and gut microbiota in high-fat-diet-induced type 2 diabetic rats. J Funct Foods. 2020;69:103939. 10.1016/j.jff.2020.103939. 10.1016/j.jff.2020.103939

[59] Beale AL, O’Donnell JA, Nakai ME, Nanayakkara S, Vizi D, Carter K, et al. The gut microbiome of heart failure with preserved ejection fraction. J Am Heart Assoc. 2021;10:e020654. 10.1161/JAHA.120.020654. 34212778 10.1161/JAHA.120.020654 PMC8403331

[60] Simadibrata DM, Auliani S, Widyastuti PA, Wijaya AD, Amin HZ, Muliawan HS, et al. The Gut Microbiota Profile in Heart failure patients: a systematic review. J Gastrointestin Liver Dis. 2023;32:393–401. 10.15403/jgld-4779. 37774217 10.15403/jgld-4779

[61] Büttner P, Werner S, Böttner J, Ossmann S, Schwedhelm E, Thiele H. Systemic effects of Homoarginine supplementation on arginine metabolizing enzymes in rats with heart failure with preserved ejection fraction. Int J Mol Sci. 2023. 10.3390/ijms241914782. 37834229 10.3390/ijms241914782 PMC10572665

[62] Chen W-S, Wang C-H, Cheng C-W, Liu M-H, Chu C-M, Wu H-P, et al. Elevated plasma phenylalanine predicts mortality in critical patients with heart failure. ESC Heart Fail. 2020;7:2884–93. 10.1002/ehf2.12896. 32618142 10.1002/ehf2.12896 PMC7524095

[63] Wang C-H, Cheng M-L, Liu M-H. Simplified plasma essential amino acid-based profiling provides metabolic information and prognostic value additive to traditional risk factors in heart failure. Amino Acids. 2018;50:1739–48. 10.1007/s00726-018-2649-9. 30203393 10.1007/s00726-018-2649-9

[64] Teunis CJ, Stroes ESG, Boekholdt SM, Wareham NJ, Murphy AJ, Nieuwdorp M, et al. Tryptophan metabolites and incident cardiovascular disease: the EPIC-Norfolk prospective population study. Atherosclerosis. 2023;387:117344. 10.1016/j.atherosclerosis.2023.117344. 37945449 10.1016/j.atherosclerosis.2023.117344 PMC12512466

[65] Melhem NJ, Taleb S. Tryptophan: from diet to cardiovascular diseases. Int J Mol Sci. 2021. 10.3390/ijms22189904. 34576067 10.3390/ijms22189904 PMC8472285

[66] Sandek A, Bjarnason I, Volk H-D, Crane R, Meddings JB, Niebauer J, et al. Studies on bacterial endotoxin and intestinal absorption function in patients with chronic heart failure. Int J Cardiol. 2012;157:80–5. 10.1016/j.ijcard.2010.12.016. 21190739 10.1016/j.ijcard.2010.12.016

[67] Li N, Zhou H, Wu H, Wu Q, Duan M, Deng W, Tang Q. STING-IRF3 contributes to lipopolysaccharide-induced cardiac dysfunction, inflammation, apoptosis and pyroptosis by activating NLRP3. Redox Biol. 2019;24:101215. 10.1016/j.redox.2019.101215. 31121492 10.1016/j.redox.2019.101215 PMC6529775

[68] Bowman JD, Surani S, Horseman MA. Endotoxin, toll-like Receptor-4, and atherosclerotic heart disease. Curr Cardiol Rev. 2017;13:86–93. 10.2174/1573403X12666160901145313. 27586023 10.2174/1573403X12666160901145313 PMC5452150

[69] Chen S-Y, Rong X-Y, Sun X-Y, Zou Y-R, Zhao C, Wang H-J. A novel trimethylamine oxide-induced model implicates gut microbiota-related mechanisms in frailty. Front Cell Infect Microbiol. 2022;12:803082. 10.3389/fcimb.2022.803082. 35360115 10.3389/fcimb.2022.803082 PMC8963486

[70] Gawrys-Kopczynska M, Konop M, Maksymiuk K, Kraszewska K, Derzsi L, Sozanski K, et al. TMAO, a seafood-derived molecule, produces diuresis and reduces mortality in heart failure rats. Elife. 2020. 10.7554/eLife.57028. 32510330 10.7554/eLife.57028 PMC7334024

[71] Zhao Y, Wang Z. Impact of trimethylamine N-oxide (TMAO) metaorganismal pathway on cardiovascular disease. J Lab Precis Med. 2020. 10.21037/jlpm.2020.01.01. 32587943 10.21037/jlpm.2020.01.01 PMC7316184

[72] Velasquez MT, Ramezani A, Manal A, Raj DS. Trimethylamine N-oxide: the good, the bad and the unknown. Toxins (Basel). 2016. 10.3390/toxins8110326. 27834801 10.3390/toxins8110326 PMC5127123

[73] Wastyk HC, Fragiadakis GK, Perelman D, Dahan D, Merrill BD, Yu FB, et al. Gut-microbiota-targeted diets modulate human immune status. Cell. 2021;184:4137–e415314. 10.1016/j.cell.2021.06.019. 34256014 10.1016/j.cell.2021.06.019 PMC9020749

[74] Jiang S, Shui Y, Cui Y, Tang C, Wang X, Qiu X, et al. Gut microbiota dependent trimethylamine N-oxide aggravates angiotensin II-induced hypertension. Redox Biol. 2021;46:102115. 10.1016/j.redox.2021.102115. 34474396 10.1016/j.redox.2021.102115 PMC8408632

[75] Taylor CR, Levenson RM. Quantification of immunohistochemistry–issues concerning methods, utility and semiquantitative assessment II. Histopathology. 2006;49:411–24. 10.1111/j.1365-2559.2006.02513.x. 16978205 10.1111/j.1365-2559.2006.02513.x

