# Supplementary material for: Interactions between the gut microbiome, associated metabolites and the manifestation and progression of heart failure with preserved ejection fraction in ZSF1 rats
Source: Cardiovasc Diabetol. 2024 Aug 14;23:299. doi: 10.1186/s12933-024-02398-6 (PMC11325580; doi:10.1186/s12933-024-02398-6)
Supplement: Supplementary file 1 — Supplementary Material 1. [file 12933_2024_2398_MOESM1_ESM.docx]

**Interactions between the gut microbiome, associated metabolites and the manifestation and progression of heart failure with preserved ejection fraction in ZSF1 rats**

Salmina J Guivala, Konrad A Bode, Jürgen G Okun, Ece Kartal, Luca Valentina Pohl, Sarah Werner, Sandra Erbs, Holger Thiele, Petra Büttner


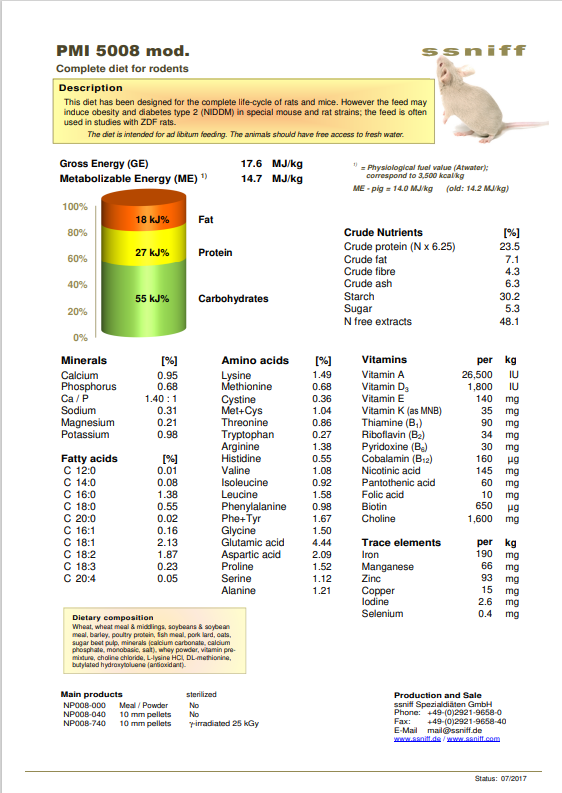


**Supplementary figure 1:** Dietary Composition of the diet 5008° (ssniff Spezialdiäten, Soest, Germany) provided to all animals. Standard chow was rich in energy and protein content. The chow contains 23% protein and 6.5% fat. Fish meal, porcine fat and meat are part of the recipe.


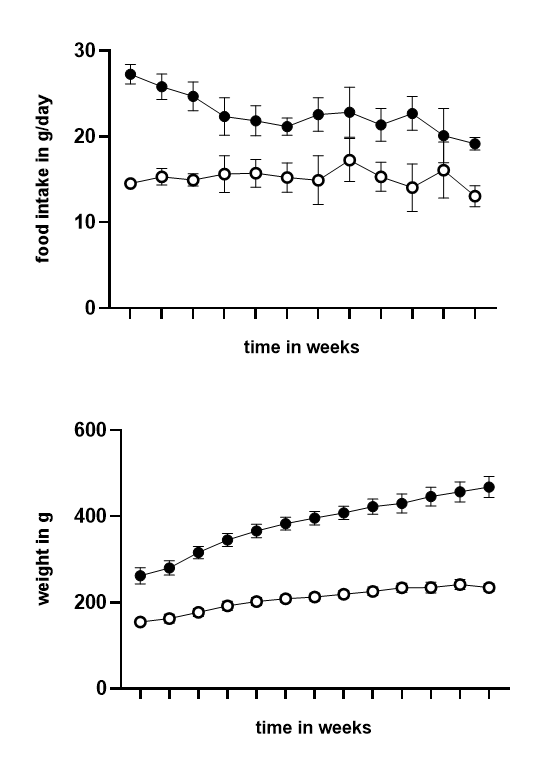


**Supplementary figure 2:** Weight (gram) (upper panel) as well as food intake (gram per day) (lower panel) of lean (white circles) and obese (black circles) ZSF1 rats. Measurements were started at 7 / 8 weeks of age and continued until 20 weeks of age.


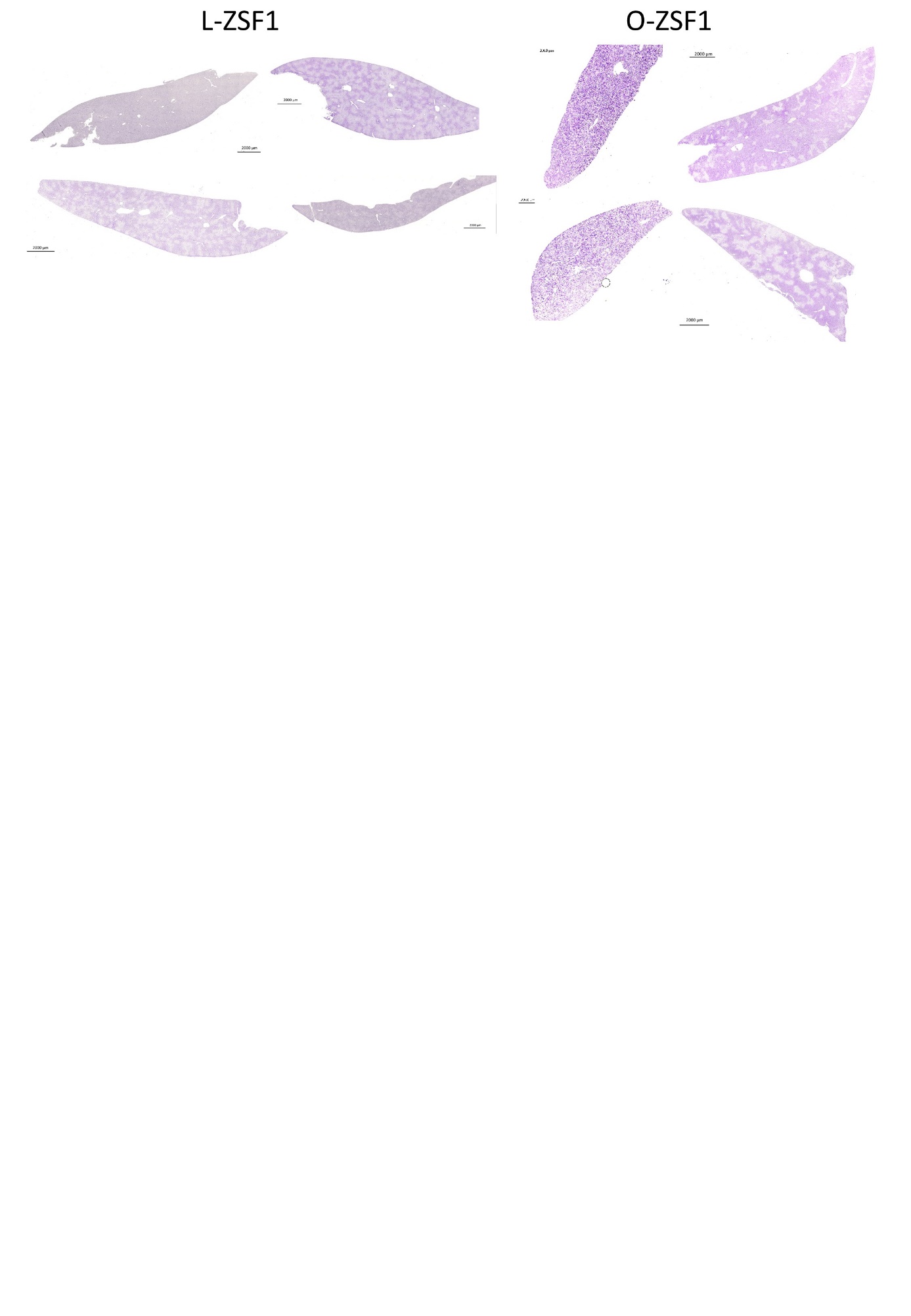


**Supplementary figure 3:** Histological analysis of ZSF1 liver glycogen content using periodic acid shift (PAS) staining. Four exemplarily individual liver stains per experimental group are presented. The PAS stains glycogen blue to violet. This was more pronounced in O-ZSF1 rats, indicating higher glycogen level.

**
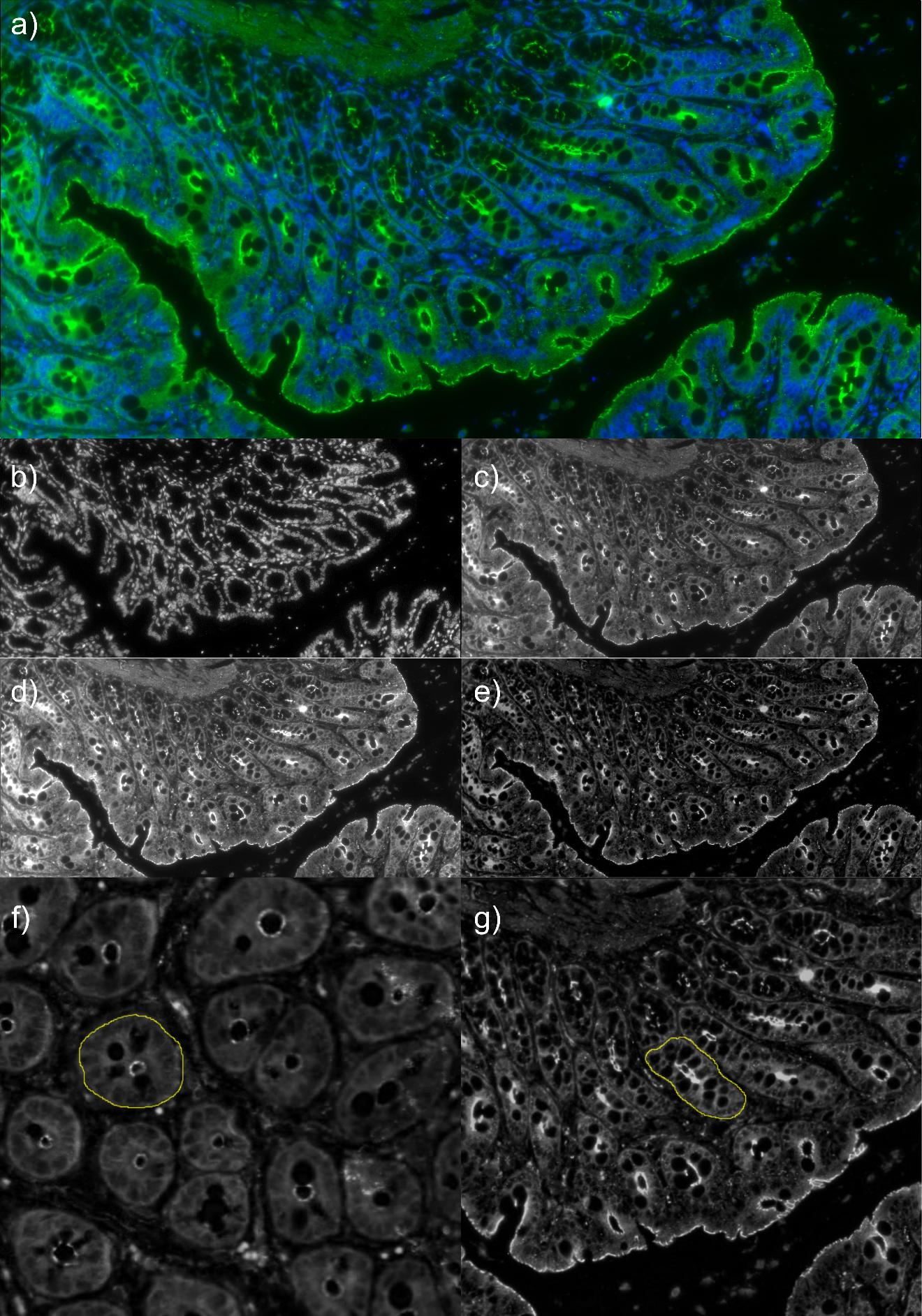
Supplementary figure 4:** Image Processing and Analysis of ZO1-antibody-staining images using Image J / Fiji. a) Immunohistochemistry image stained with ZO1-antibodies in a colon sample of a lean ZSF1 rat, b) 8-bit image of the blue channel and c) 8-bit image of the green changel after splitting channels, d) green channel image after automatically adjusting brightness & contrast, e) the same image after subtracting background noise using a rolling ball radius of 50 pixel, f) and g) adjusted images showing colon crypts circled as regions of interest


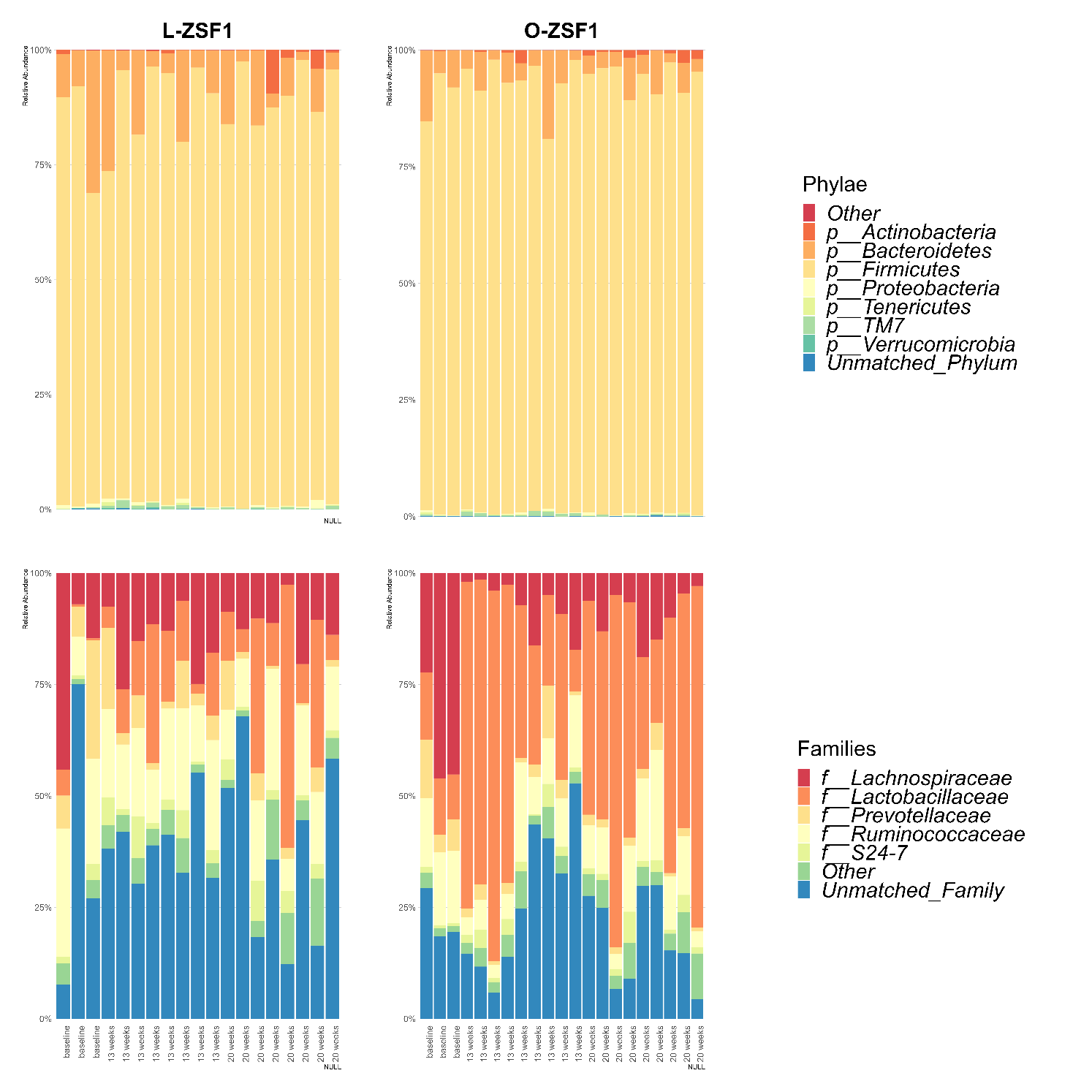


**Supplementary Figure** **5: Upper panels** - Phylum-level core microbiome composition for individual samples over time in lean (L-ZSF1) and obese (O-ZSF1) animals determined at eight (baseline), 13 and 20 weeks using relative abundance of phylae. Taxa were aggregated on the phylum-level using a detection threshold of 0.001 and a prevalence threshold of 0.1. **Lower panels -** Family-level core microbiome composition for individual samples over time in lean and obese animals at eight, 13 and 20 weeks using relative abundance of families and portraying the top five most prevalent families. Taxa were aggregated on the family-level using a detection threshold of 0.01 and a prevalence threshold of 0.5.

**
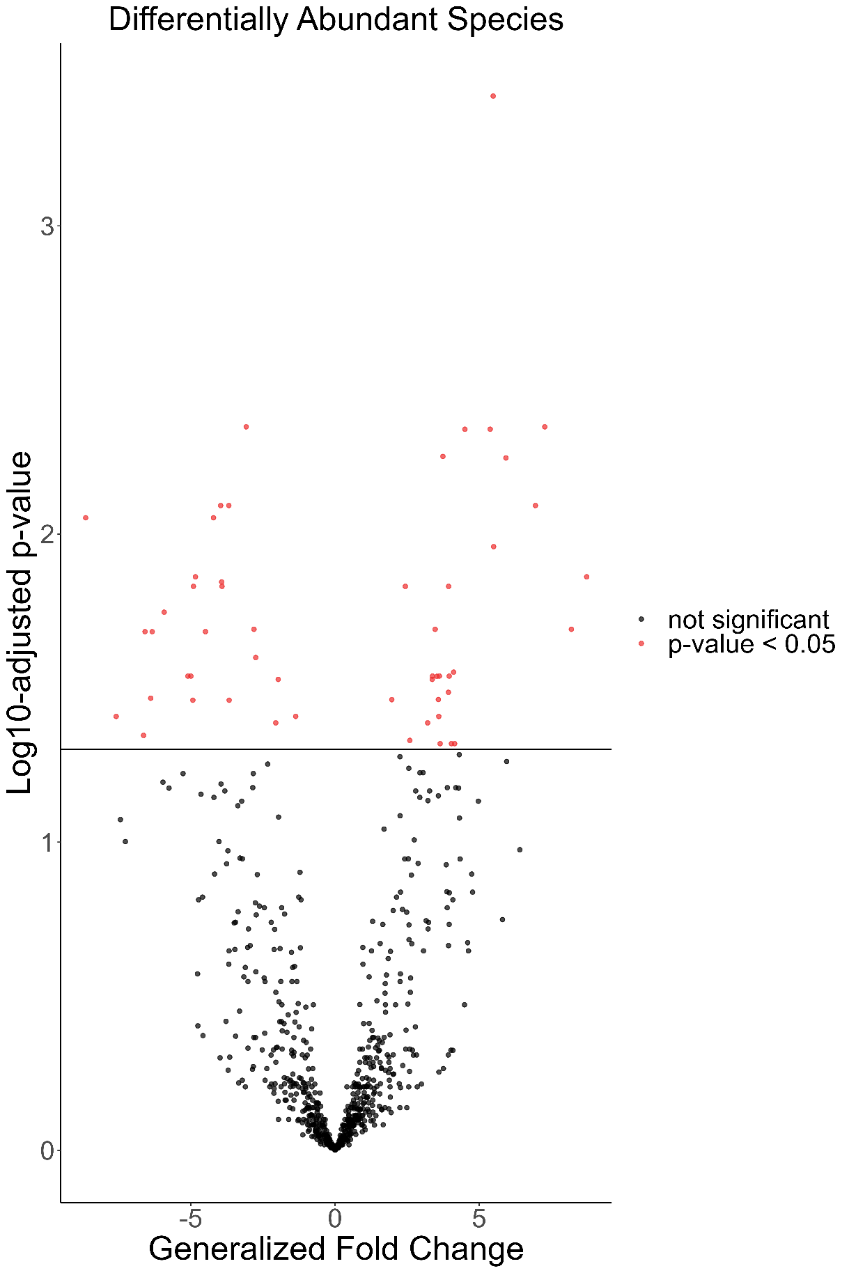
**

**Supplementary figure 6:** Differential abundance of different species comparing lean and obese ZSF1 rats at 20 weeks of age. Generalized Fold Change is given in Log2-Fold-Change as a parameter of differences in species expression between the two conditions. P-values were log10-transformed for better visualization.

| **Parameter** | **O-ZSF1**  **(mean±STD)** | **L-ZSF1**  **(mean±STD)** | **p-value** |
| --- | --- | --- | --- |
| LVEF (M-Mode) | 63±15 % | 55±12 % | 0.140 |
| LVESV | 0.428 ± 0.106 mL | 0.333 ± 0.039 mL | 0.013 |
| LVEDV | 0.598 ± 0.111 mL | 0.461 ± 0.040 mL | 0.001 |
| E/e’ septal | 16.199 ± 2.522 | 12.203 ± 1.610 | < 0.001 |
| E/e’ lateral | 21.720 ± 3.570 | 15.003 ± 2.782 | < 0.001 |
| TAPSE | 3.182 ± 0.462 mm | 2.833 ± 0.42 mm | 0.100 |

**Supplementary table 1:** Echocardiography data of lean and obese ZSF1 rats at 20 weeks of age acquired using noninvasive transthoracic echocardiography (Vivid-J, GE Healthcare, Chicago, USA).

| **Phylum** | **Class** | **Order** | **Family** | **Genus [species]** | **log2FC** | **padj** |
| --- | --- | --- | --- | --- | --- | --- |
| F | Clostridia | Clostridiales | Ruminococcaceae | Ruminococcus | -2.4 | 0.000 |
| F | Clostridia | Clostridiales |  |  | . | 0.000 |
| B | Bacteroidia | Bacteroidales | S24-7 |  | -3.1 | 0.004 |
| F | Clostridia | Clostridiales | Ruminococcaceae |  | 7.3 | 0.004 |
| F | Clostridia | Clostridiales | Lachnospiraceae |  | 4. | 0.00 |
| F | Clostridia | Clostridiales |  |  | .4 | 0.00 |
| F | Clostridia | Clostridiales |  |  | 3.7 | 0.006 |
| F | Clostridia | Clostridiales | Ruminococcaceae | Oscillospira | .9 | 0.006 |
| F | Clostridia | Clostridiales | Lachnospiraceae | Ruminococcus [gnavus] | -4.0 | 0.008 |
| F | Clostridia | Clostridiales |  |  | 7.0 | 0.008 |
| F | Bacilli | Lactobacillales | Lactobacillaceae | Lactobacillus | -3.7 | 0.008 |
| F | Clostridia | Clostridiales |  |  | -8.7 | 0.009 |
| F | Erysipelotrichi | Erysipelotrichales | Erysipelotrichaceae | Allobaculum | -4.2 | 0.009 |
| F | Clostridia | Clostridiales | Ruminococcaceae | Ruminococcus | . | 0.011 |
| F | Clostridia | Clostridiales |  |  | -4.8 | 0.014 |
| F | Clostridia | Clostridiales |  |  | 8.7 | 0.014 |
| F | Clostridia | Clostridiales | Lachnospiraceae |  | -3.9 | 0.014 |
| F | Clostridia | Clostridiales |  |  | 3.9 | 0.01 |
| F | Clostridia | Clostridiales | Lachnospiraceae | Ruminococcus [gnavus] | 2.4 | 0.01 |
| F | Bacilli | Lactobacillales | Lactobacillaceae | Lactobacillus | -3.9 | 0.01 |
| F | Clostridia | Clostridiales |  |  | -4.9 | 0.01 |
| F | Clostridia | Clostridiales | Lachnospiraceae | Coprococcus | -.9 | 0.018 |
| F | Clostridia | Clostridiales | Ruminococcaceae | Oscillospira | 3. | 0.020 |
| B | Bacteroidia | Bacteroidales | S24-7 |  | -2.8 | 0.020 |
| F | Clostridia | Clostridiales |  |  | 8.2 | 0.020 |
| F | Clostridia | Clostridiales | Lachnospiraceae | Blautia | -6.6 | 0.021 |
| F | Clostridia | Clostridiales |  |  | -4. | 0.021 |
| F | Clostridia | Clostridiales |  |  | -6.3 | 0.021 |
| F | Bacilli | Lactobacillales | Lactobacillaceae | Lactobacillus | -2.7 | 0.02 |
| F | Clostridia | Clostridiales |  |  | 4.1 | 0.028 |
| F | Clostridia | Clostridiales |  |  | 3.4 | 0.029 |
| F | Clostridia | Clostridiales | Ruminococcaceae | Oscillospira | 4.0 | 0.029 |
| F | Clostridia | Clostridiales |  |  | -.0 | 0.029 |
| F | Clostridia | Clostridiales | Lachnospiraceae |  | 3.6 | 0.029 |
| F | Clostridia | Clostridiales |  |  | 3. | 0.029 |
| F | Bacilli | Lactobacillales | Lactobacillaceae | Lactobacillus | -2.0 | 0.030 |
| F | Clostridia | Clostridiales |  |  | 3.4 | 0.030 |
| F | Clostridia | Clostridiales | Ruminococcaceae | Ruminococcus | 3.9 | 0.033 |
| F | Clostridia | Clostridiales |  |  | -6.4 | 0.034 |
| F | Clostridia | Clostridiales | Ruminococcaceae | Oscillospira | 3.6 | 0.034 |
| F | Clostridia | Clostridiales |  |  | 2.0 | 0.034 |
| F | Clostridia | Clostridiales |  |  | -4.9 | 0.03 |
| F | Clostridia | Clostridiales | Lachnospiraceae | Ruminococcus [gnavus] | -3.7 | 0.03 |
| F | Bacilli | Lactobacillales | Lactobacillaceae | Lactobacillus [reuteri] | -1.4 | 0.039 |
| F | Clostridia | Clostridiales | Lachnospiraceae |  | 3.6 | 0.039 |
| F | Clostridia | Clostridiales |  |  | -7.6 | 0.039 |
| B | Bacteroidia | Bacteroidales | Rikenellaceae |  | -2.1 | 0.041 |
| F | Clostridia | Clostridiales |  |  | 3.2 | 0.041 |
| F | Clostridia | Clostridiales | Lachnospiraceae | Coprococcus | -6.6 | 0.04 |
| F | Clostridia | Clostridiales | Ruminococcaceae | Oscillospira | 2.6 | 0.047 |
| F | Clostridia | Clostridiales | Lachnospiraceae |  | 4.1 | 0.048 |
| F | Clostridia | Clostridiales |  |  | 3.6 | 0.048 |
| F | Clostridia | Clostridiales | Ruminococcaceae | Ruminococcus | 4.0 | 0.048 |

**Supplementary Table 2:** Differential Abundance Level at 20 weeks of age. Phylum: F=Firmicutes, B=Bacteroidetes, FC=fold change, Genus [species] – when the species was identified the information is given as an extension of the genus in brackets. Padj – P-value adjusted for multiple testing.

**Supplementary Information on the R-Packages that were used in this study**

*“cowplot” -* Wilke C (2020). _cowplot: Streamlined Plot Theme and Plot Annotations for 'ggplot2'_. R package version 1.1.1, <https://CRAN.R-project.org/package=cowplot>.

*“DESeq2” -* Love, Michael I.; Huber, Wolfgang; Anders, Simon (2014): Moderated estimation of fold change and dispersion for RNA-seq data with DESeq2. In: Genome biology 1 (12), S. 0. DOI: 10.1186/s1309-014-00-8.

*“ggplot2” -* Wickham, Hadley (2016): ggplot2. Elegant Graphics for Data Analysis. 2nd ed. 2016. Cham: Springer International Publishing; Imprint: Springer (Use R!).

*“gridExtra” - Auguie B (2017). _gridExtra: Miscellaneous Functions for "Grid" Graphics_. R package version 2.3, <**https://CRAN.R-project.org/package=gridExtra>.*

*“microbiome” -* Leo Lahti et al. (2012-2019): microbiome R package. <https://microbiome.github.io>

*“phyloseq” -* McMurdie, Paul J.; Holmes, Susan (2013): phyloseq: an R package for reproducible interactive analysis and graphics of microbiome census data. In: *PloS one* 8 (4), e61217. DOI: 10.1371/journal.pone.0061217.

*“vegan” -* Oksanen J, Simpson G, Blanchet F et al. (2022): _vegan: Community Ecology Package_. R package version 2.6-4, <https://CRAN.R-project.org/package=vegan>
